# The differential impact of *HNRNPA1* isoforms on gene expression and their relevance to dsRNA-mediated innate immune response

**DOI:** 10.1038/s41598-025-99031-7

**Published:** 2025-05-01

**Authors:** Jade-Emmanuelle Deshaies, Valérie Triassi, Andréanne Lacombe, Myriam Gagné, Karen Ling, Asmita Ghosh, Marjorie Labrecque, Frank Rigo, Paymaan Jafar-nejad, Martine Tétreault, Christine Vande Velde

**Affiliations:** 1 Centre hospitalier de l’Université de Montréal (CHUM) Research Center, Montréal, QC Canada; 2 Department of Neurosciences, Université de Montréal, Montréal, QC Canada; 3 Department of Biochemistry and Molecular Medicine, Université de Montréal, Montréal, QC Canada; 4 Department of Core Antisense Research, Ionis Pharmaceuticals, Inc, Carlsbad, CA USA; 5 Department of Neurosciences, Université de Montréal CRCHUM-Tour Viger, 900, rue Saint-Denis, R09.474, Montreal, QC H2X 0A9 Canada

**Keywords:** *HNRNPA1*, Isoforms, RNA sequencing (RNA-seq), Differential gene expression, Double-stranded RNA, Innate immunity, Transcriptomics, Cell biology

## Abstract

Heterogeneous nuclear ribonucleoprotein A1 (*HNRNPA1*) is a highly abundant RNA binding protein alternatively spliced in two main isoforms named, hnRNP A1 and hnRNP A1B. While being ubiquitously expressed, both isoforms have different cellular localizations and are differentially expressed in tissues during development and aging. To improve our understanding of the cellular function of each isoform, we performed RNA sequencing in cells exclusively expressing hnRNP A1 or hnRNP A1B. As expected, some genes were commonly regulated, however > 300 genes were differentially regulated by the two isoforms. Functional annotation indicated an enrichment for genes implicated in cellular defense, especially for innate immunity and dsRNA response. Here, we demonstrate that in basal conditions, hnRNP A1, but not hnRNP A1B, represses interferon stimulated genes including the family of dsRNA sensors oligoadenylate synthases (OASs). Thus, the dsRNA-mediated interferon antiviral response can be potentiated by the loss of hnRNP A1-mediated repression.

**Supplementary Information:**

The online version contains supplementary material available at 10.1038/s41598-025-99031-7.

## Introduction

Heterogeneous nuclear ribonucleoprotein A1 (hnRNP A1) is the most abundant RNA binding protein (RBP) in the hnRNP(A/B) subfamily^1^. Its capacity to broadly bind RNA and DNA defines it as an important factor in RNA metabolism and thus an important player in a broad range of cellular processes^2,3^. Indeed, hnRNP A1 has been implicated in transcriptional regulation, telomere maintenance, RNA splicing, miRNA biogenesis, protein translation and mRNA transport^3,4^. The mammalian gene *HNRNPA1* can be spliced into multiple transcripts^5,6^, however it encodes two main protein coding variants which differ by the exclusion (transcript variant 1, hnRNP A1) or inclusion (transcript variant 2, hnRNP A1B) of the cassette exon 7B. The N-terminus of both isoforms contains two non-redundant RNA recognition motifs (RRM) able to bind distinct RNA and mRNA sequences^7,8^. The C-terminus consists of an intrinsically disordered region (IDR) composed of a glycine-rich region that includes an RGG-box and the M9 domain, the latter of which dictates nucleocytoplasmic shuttling^3,4,9^. The inclusion of exon 7B adds 52 amino acids to the glycine-rich region^10^, a domain which is considered important for protein-protein interactions, subcellular localization and RNA/DNA binding. We have also previously shown that the inclusion of this exon increases its propensity to fibrillate and hence aggregate^11^.

It has been demonstrated that non-mammalian vertebrates express only the longer *HNRNPA1* isoform, with exon 7B being a constitutive exon in non-mammals^12^. The emergence of the second isoform is due to the introduction of an intronic sequence (conserved element 6, CE6) located downstream of exon 7B. CE6 effectively masks the 5’splice site resulting in exon 7B skipping^12,13^. Emergence of new long range RNA-RNA interactions promoting alternative splice events is common in the hnRNP A and D family, suggesting that they are of functional importance^12,14^. We have previously reported that hnRNP A1 isoforms are differentially expressed in mouse tissues, with the hnRNP A1B isoform being predominantly expressed in the central nervous system (CNS)^9,15,16^. As new evidence of *HNRNPA1* regulation emerges^11,17^, we are only now starting to unveil its complex function which is often context-specific. Even though *HNRNPA1* is ubiquitously expressed, changes in protein levels and localization can have variable consequences depending on the cell type and stimulus^18–20^. Indeed, *HNRNPA1* has been implicated in a broad range of pathologies including autoimmune disease, neurodegenerative disease, myopathy, and cancer^3,10,11,17,21–24^. Moreover, it is also highly modulated during viral infection where hnRNP A1 can either enhance viral replication and survival or confer a protective response^1^.

Here, we performed transcriptomics analyses to dissect the contribution of each isoform to gene expression and found that both isoforms share the regulation of many genes but also have isoform-specific targets. Both isoforms impact a variety of cellular and metabolic processes but seem unable to fully functionally replace for one other. We also demonstrate that hnRNP A1, but not hnRNP A1B, is responsible for the repression of a subset of innate immunity genes in basal conditions, including members of the oligoadenylate synthase (OAS) family which are sensors of dsRNA. Our data indicate that the loss of hnRNP A1, but not hnRNP A1B, increases the activity of an effector of the dsRNA-mediated antiviral interferon response protein kinase R (PKR), resulting in increased interferon expression.

## Results

### *HNRNPA1* isoforms regulate a common gene set as well as isoform-specific genes

**Fig. 1:**
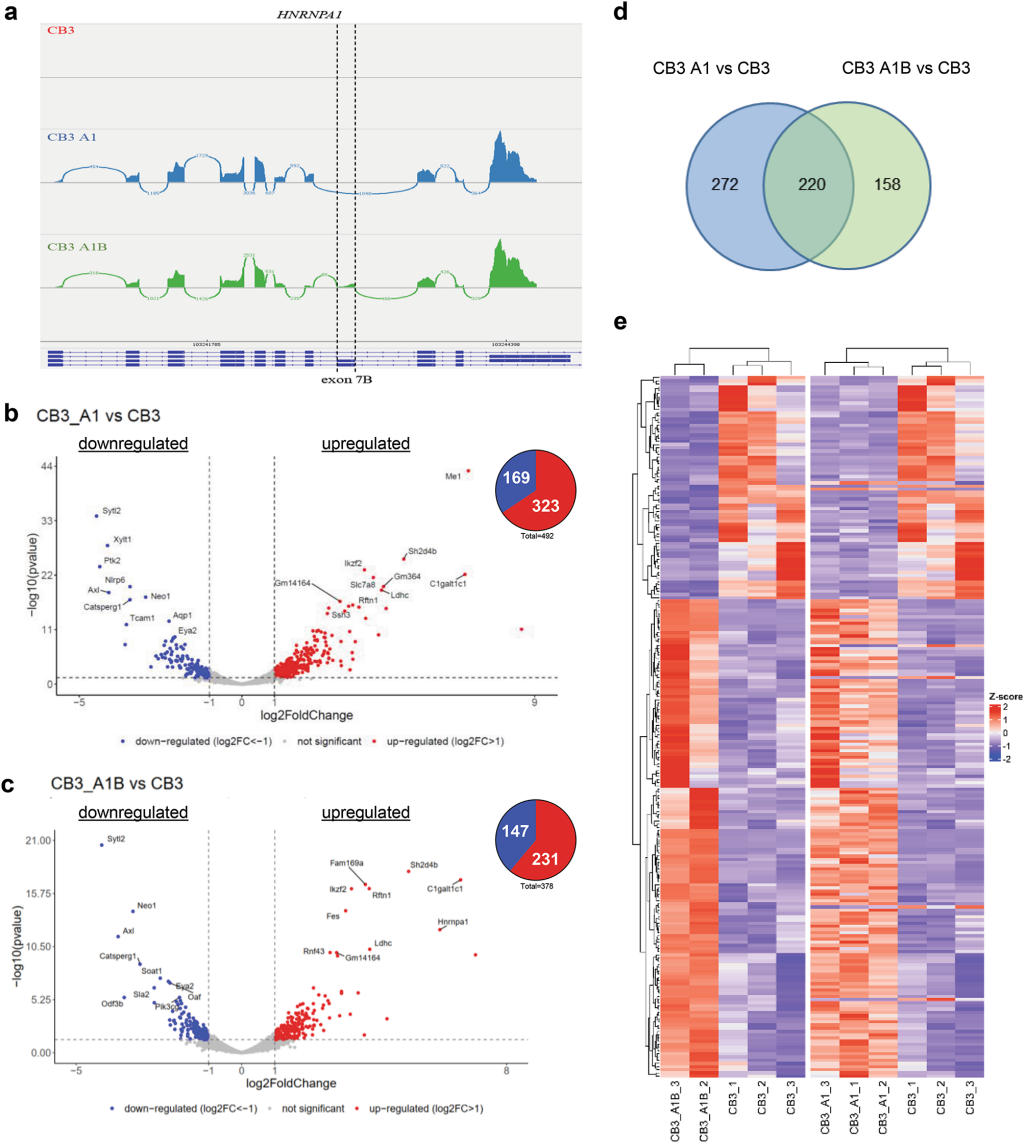
Exclusive expression of HNRNPA1 isoforms regulated a common set of genes and an isoform-specific pool of genes. (**A**) Sashimi plot for *HNRNPA1*. (**B**,**C**) Volcano plots of the differentially expressed genes (DEGs) and pie chart for upregulated and downregulated genes in the CB3 A1 vs. CB3 comparison (**B**) and in the CB3 A1B vs. CB3 comparison (**C**). (**D**) Venn diagram showing the number of genes that were differentially expressed with the expression of either one of the isoforms or commonly regulated by both. (**E**) Heatmap of DEGs in both comparisons.

To individually evaluate the impact of *HNRNPA1* isoforms on the transcriptome, we took advantage of the mouse erythroleukemia cell line CB3 in which the endogenous *HNRNPA1* locus has been inactivated, and two derivative cell lines where hnRNP A1 and hnRNP A1B expression have been separately reconstituted to comparable levels, referred to as CB3 A1 and CB3 A1B, respectively^11,25^. In these cells, *HNRNPA1* transcript and protein levels were confirmed by qRT-PCR and immunoblotting, respectively (Fig. S1A,B). Total RNA, depleted of ribosomal RNA (rRNA), from each cell line was processed for RNA-sequencing (RNA-Seq) and a comparative analysis of gene expression using DESeq2^26^ was performed (Fig. S1C, Supp. Table 1, Supp. Data File 1). Visualization of RNA-Seq reads with sashimi plots confirmed the similar expression levels of protein-encoding *HNRNPA1* transcripts in both CB3 A1 and CB3 A1B cell lines, as well as confirmed that CB3 cells are effectively a knockout for *HNRNPA1* (Fig. 1A). Principal component analysis (PCA) demonstrates that the gene expression profiles of the different cell lines were separated by the first component, placing CB3 A1B in between the CB3 A1 and CB3 profiles (Fig. S1D).

To evaluate the effect of the exclusive expression of *HNRNPA1* isoforms on the transcriptome, we assessed differential gene expression comparing CB3 A1 versus CB3 and CB3 A1B versus CB3 using a p-value < 0.05 and a Log2FoldChange ≥ 1 as a significant threshold. Expression of hnRNP A1 compared to the parental line significantly influenced the expression of 492 genes (323 upregulated, 169 downregulated; Fig. 1B), while the expression of hnRNP A1B impacted 378 genes (231 upregulated, 147 downregulated; Fig. 1C). We compared the list of genes differentially expressed by either hnRNP A1 or hnRNP A1B expression and found that both isoforms jointly regulated 220 genes, while 272 and 158 genes were significantly changed with exclusive hnRNP A1 or hnRNP A1B expression, respectively (Fig. 1D, Supp. Data File 2). It was noted that when compared to the parental CB3 cell line, genes determined to be differentially expressed in CB3 A1 and CB3 A1B samples were typically changed in the same direction (Fig. 1E). Collectively, these findings suggest that the *HNRNPA1* isoforms regulate distinct gene sets in addition to a common gene set, suggesting that both are required for cellular homeostasis.

### HnRNP A1 and HnRNP A1B modulate common and isoform-specific cellular functions

**Fig. 2:**
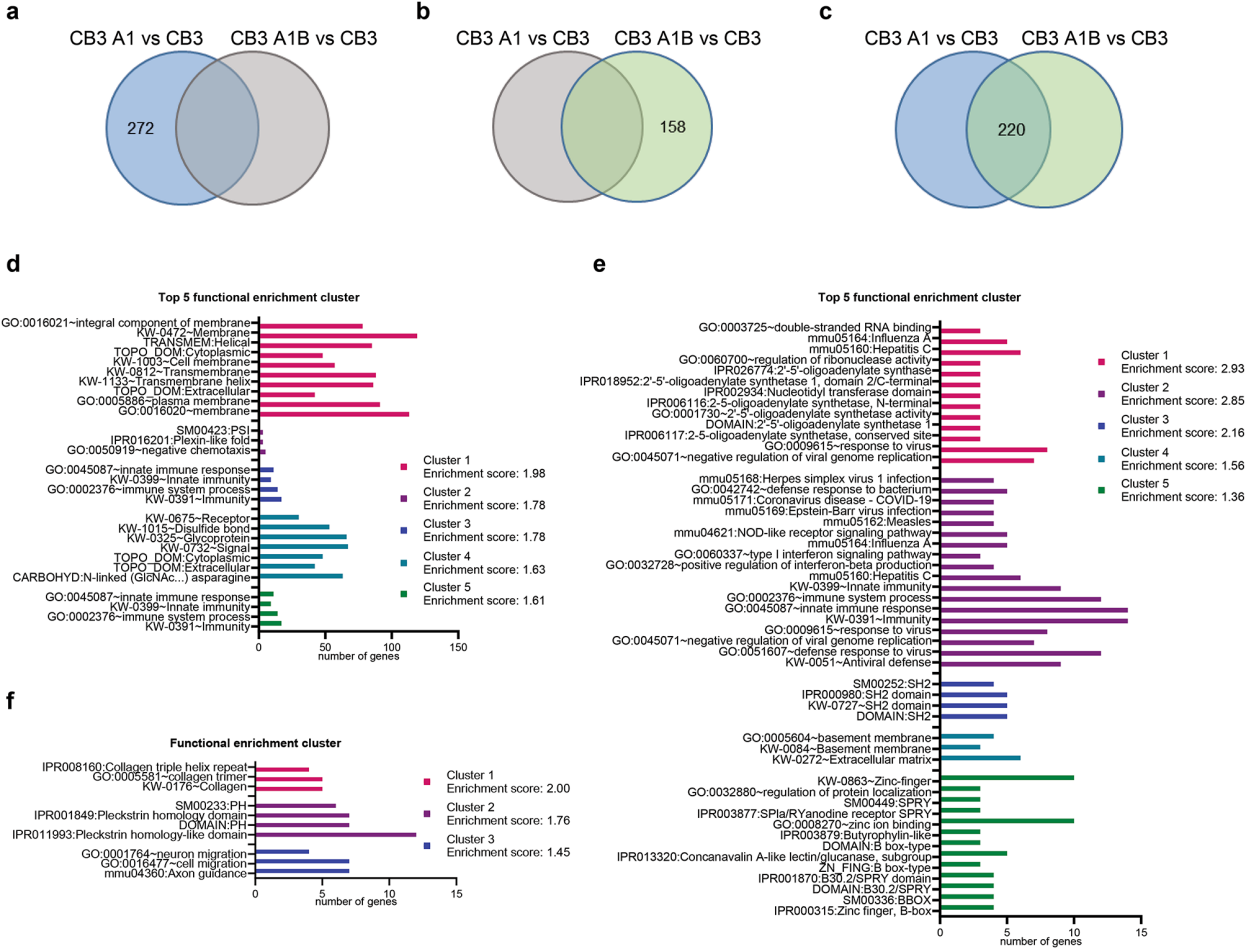
GO-term analysis of genes that are specifically regulated by the expression of only one of the isoforms or both. GO-term analysis (top 5 pathways) of genes identified (in Fig. 1D) to be differentially regulated only in (**A**,**D**) CB3 A1 vs. CB3, (**B**,**E**) only in CB3 A1B vs. CB3, or (**C**,**F**) in both comparisons using DAVID functional annotation clustering with an enrichment score > 1.3.

In an effort to understand how the observed differences in gene expression could impact cellular function, we performed gene ontology (GO) analysis using DAVID functional clustering^27^. Clusters with an enrichment score > 1.3 (-log (0.05) = 1.3) were considered significant. Using the full list of genes differentially expressed in CB3 A1 cells compared to CB3 cells, we found enrichment for 8 clusters including *immunity*,* transmembrane receptor*,* pleckstrin domain*,* calmodulin binding*,* actin binding*,* immunoglobulin domain*,* carbohydrate/carboxylic acid metabolism and lactate/malate dehydrogenase*, and *chemotaxis.* (Top 5 Fig. S2A, Supp. Data File 2). Genes differentially expressed in CB3 A1B cells compared to CB3 cells were enriched for 18 clusters including *immunity*, *pleckstrin domain*,* actin binding*,* interferon response*,* extracellular component*,* zinc-finger domain*, and *phosphatase and calmodulin-binding* (Top 5 Fig. S2B, Supp. Data File 2). To gain a better view of which cellular functions or locations were common or isoform-specific, we applied the same DAVID functional clustering approach to those genes presented in Fig. 1D. Functional clustering for the genes differentially regulated only when hnRNP A1 is expressed (compared to CB3 cells) shows an enrichment for *transmembrane protein*, *chemotaxis*,* immunity*,* membrane signalisation* and *immunoglobulin* descriptors (Top 5 Fig. 2A, D, Supp. Data File 2). In contrast, genes differentially regulated in cells having exclusive hnRNP A1B expression were enriched for *viral response*,* SH2 domain*,* basement membrane*,* SPRY domain* and *GTP binding* (Top 5 Fig. 2B, E, Supp. Data File 2). Finally, the genes that were differentially regulated by both hnRNP A1 and hnRNP A1B were enriched for *collagen*, *pleckstrin domain*, and *cell/neuron migration* (Fig. 2C, F, Supp. Data File 2). These analyses indicate that each isoform impacts a variety of cellular and metabolic processes, some of which are shared, and suggests that neither isoform is sufficient to fully functionally compensate for the other.

### HnRNP A1 and HnRNP A1B differentially regulate genes related to innate immunity

**Fig. 3:**
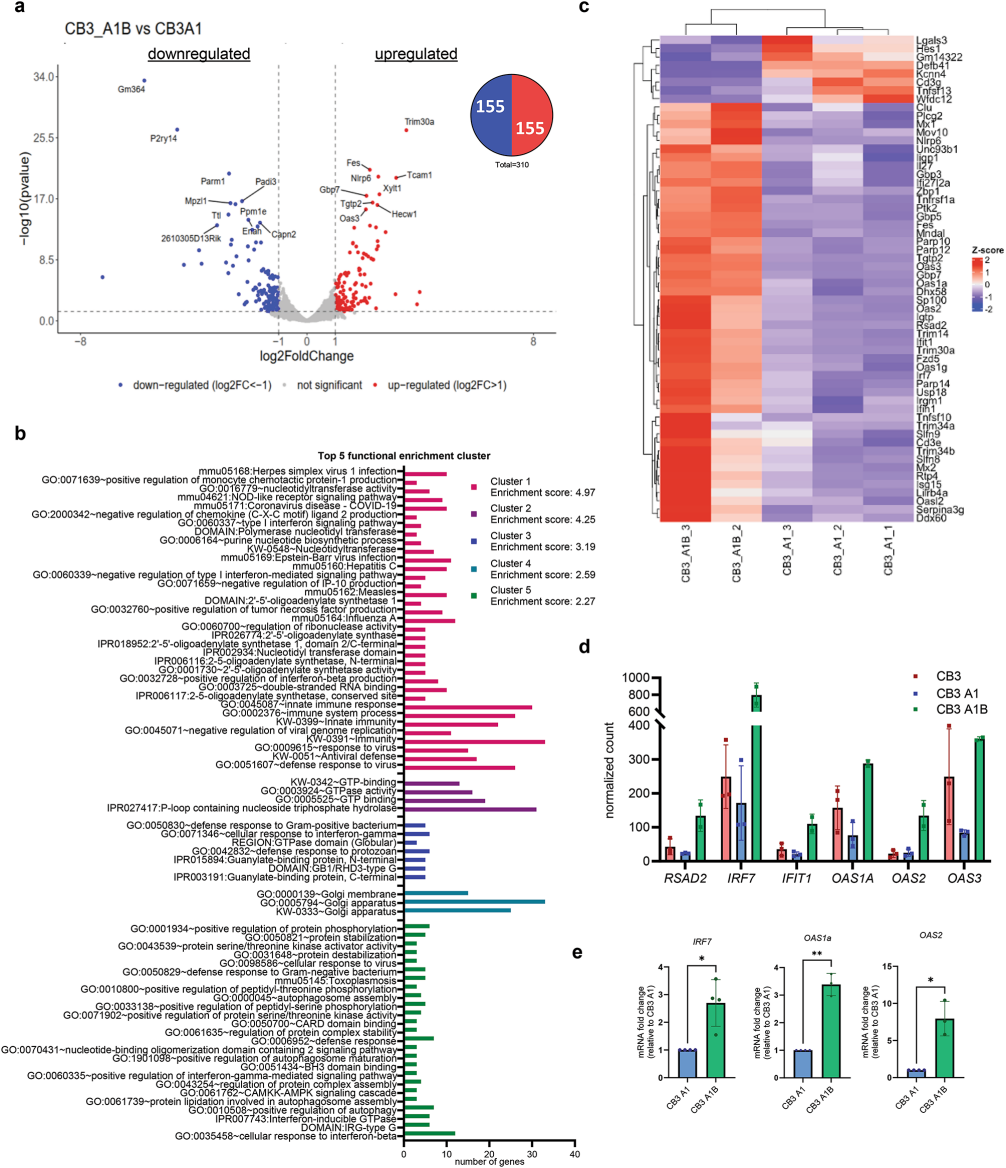
hnRNP A1 and hnRNP A1B differentially regulate genes related to innate immunity. (**A**) Volcano plot of the differentially expressed genes and pie chart of the upregulated and downregulated genes in the CB3 A1B vs. CB3 A1 comparison. (**B**) DAVID functional annotation clustering for enriched GO-term using an enrichment score > 1.3 as threshold (Top 5). (**C**) Heat map for genes found in cluster 1, innate immunity. (**D**) Normalized counts of selected interferon stimulated gene (ISG) from RNA-seq of CB3, CB3 A1 and CB3 A1B cells. (**E**) qRT-PCR for *IRF7*, *OAS1a* and *OAS2* mRNA levels in CB3 A1 vs. CB3 A1B.

We decided to directly compare the CB3 A1B and CB3 A1 datasets to better define which transcripts are significantly different when either isoform is exclusively expressed. By this approach, we found that 310 genes that were differentially regulated: 155 upregulated, 155 downregulated (Fig. 3A). Functional clustering analysis for gene ontology of the differentially expressed genes attributed to specific isoform expression indicated an enrichment for 14 clusters, with GO-terms related to *innate immunity* showing the highest enrichment score (4.97) suggesting that hnRNP A1 and hnRNP A1B differentially regulate genes relevant to innate immunity, particularly in the context of viral infection and dsRNA response (Top 5 Fig. 3B, Supp. Data File 2). Upon closer inspection of the genes included in the innate immunity cluster, we recognized that most of these genes were downregulated in hnRNP A1-expressing cells compared to the parental CB3 cells and the hnRNP A1B-expressing CB3 cells (Fig. 3C). Since many of the genes are considered as interferon stimulated genes^28^, we confirmed that this observation was not due to a differential expression of Toll-like receptors (TLRs), including TLR3 which is responsible for viral dsRNA recognition^29^ (data not shown). Given these results, we proposed that in basal conditions, hnRNP A1, but not hnRNP A1B, represses the expression of genes that contribute to innate immunity and, more precisely, to the dsRNA response. For further validation, we selected *OAS1a* (*OAS1* in *Homo sapiens*), *OAS2*, *OAS3* [all three are IFN-induced enzyme activators of Ribonuclease L (RNase L)], as well as *IRF7* (transcriptional factor of type-I interferons, IFN-I)^30^, and the interferon-inducible genes (ISG) *RSAD2* and *IFIT1* which both play important roles in the antiviral response. A closer look at the normalized count showed a lower expression of these genes in CB3 A1 compared to CB3 and to CB3 A1B (Fig. 3D). We also validated by qRT-PCR that the levels of *IRF7*, *OAS1a* and *OAS2* mRNA are significantly higher in the CB3 A1B compared to the CB3 A1 (Fig. 3E).

**Fig. 4:**
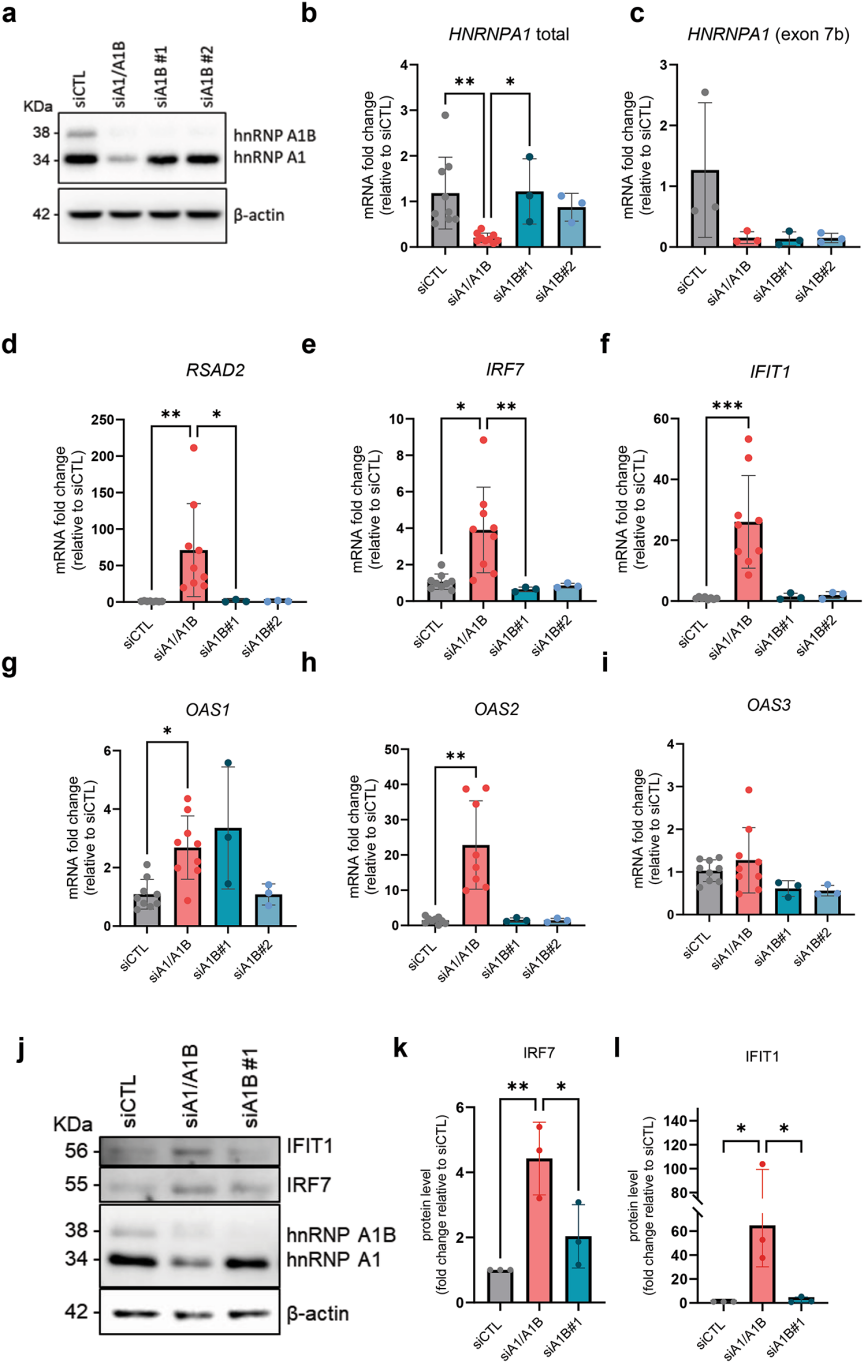
Depletion of hnRNP A1/A1B, but not hnRNP A1B, increases the level of ISGs relevant for dsRNA response in A549 cells. (**A**) Western blot for hnRNP A1, hnRNP A1B and actin. qRT-PCR showing efficient depletion of (**B**) total *HNRNPA1* or (**C**) *HNRNPA1* (exon 7b) in siA1/A1B, siA1B#1 and siA1B#2 treated cells compared to siCTL cells. (D-I) qRT-PCR for selected ISGs; (**D**) *RSAD2*, (**E**) *IRF7*, (**F**) *IFIT1*, (**G**) *OAS1*, (**H**) *OAS2*, and (**I**) *OAS3*. (**J**) Western blot for IFIT1 and IRF7 with siRNA conditions and (K-L) quantification. Kruskal–Wallis test was used for statistical significance.

To extend our results to a human context, we used the human adenocarcinoma alveolar basal epithelial cell line A549 since it is often used to evaluate molecular consequences of viral infection and of the dsRNA stress response. These cells were treated with siRNAs targeting *HNRNPA1* to effectively reduce the expression of either both isoforms (siA1/A1B) or uniquely hnRNP A1B via two independent siRNAs (siA1B #1/#2) (Fig. 4A–C). (Note, no unique sequence exists to solely reduce hnRNP A1 expression.) Compared to siControl (siCTL)-treated cells, siA1/A1B, but not siA1B treatment alone, resulted in a significant increase in the expression of *RSAD2*, *IRF7*, *IFIT1*, *OAS1*, and *OAS2* by qRT-PCR (Fig. 4D–H). However, *OAS3* was not significantly changed in A549 cells treated with siA1/A1B despite being modulated in the murine cell lines, possibly reflecting species and/or cell type differences (Fig. 4I). Elevated protein levels for IFIT1 and IRF7 in siA1/A1B condition compared to siCTL and siA1B confirmed these results (Fig. 4J-L).

**Fig. 5:**
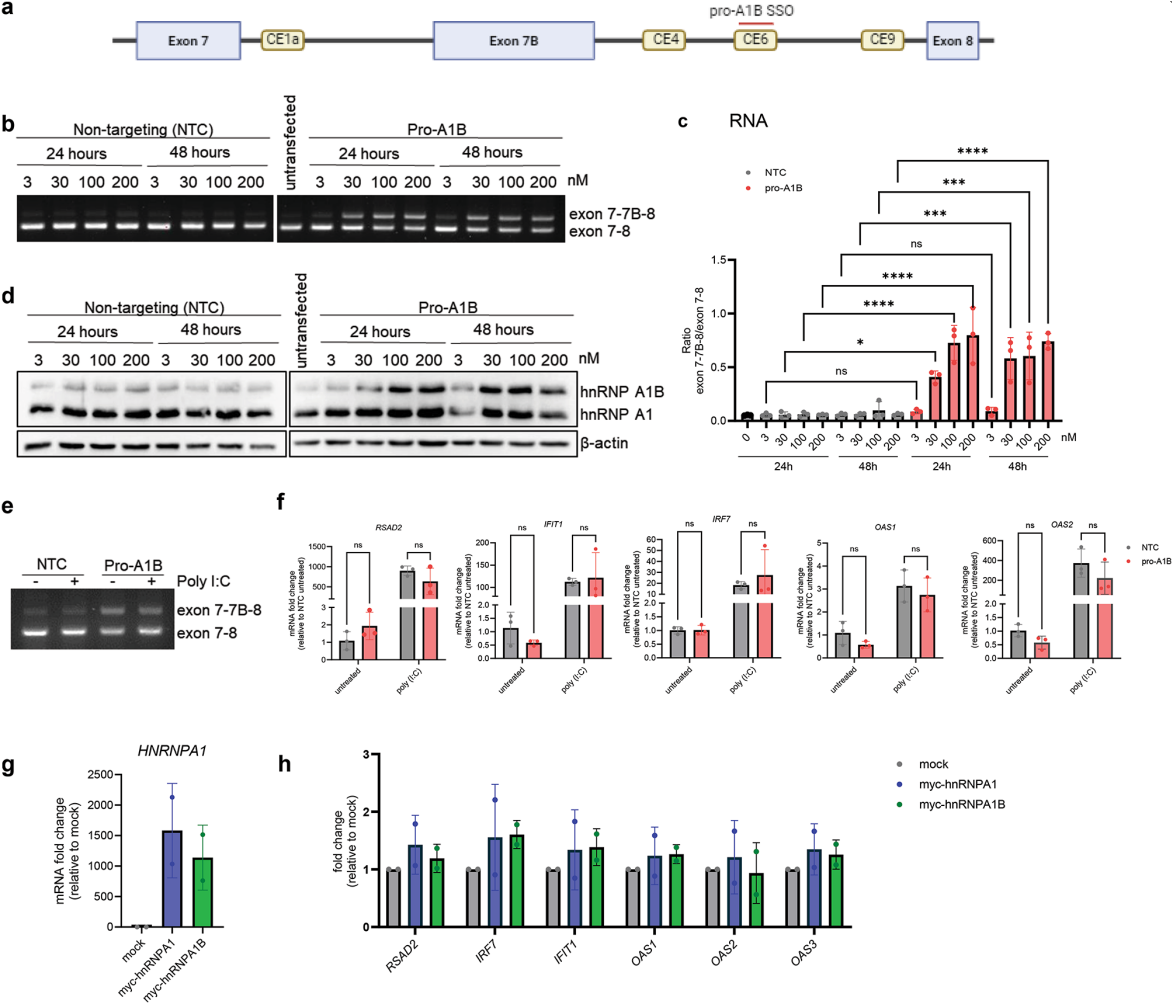
*HNRNPA1* isoform ratio does not modify the levels of innate immunity genes. (**A**) Schematic of *HNRNPA1* pre-mRNA transcript (exon 7 to 8) with location of the pro-A1B SSO. Created in BioRender. Vande Velde, C. (2025) https://BioRender.com/a65n920. (**B**) PCR for *HNRNPA1* (primers located in exon 7 and 8) in untransfected cells and cells treated with NTC or pro-A1B SSO for 24 and 48 h at 3, 30, 100 and 200 nM. (**C**) Exon 7-7B-8 / exon 7–8 was calculated and plotted. Ordinary one-way ANOVA was used for statistical analysis. (**D**) Western blot for hnRNP A1, hnRNP A1B and β-actin in untransfected cells and cells treated with NTC or pro-A1B SSO for 24 and 48 h at 3, 30, 100 and 200 nM. (**E**) RT-PCR for *HNRNPA1* and (**F**) qRT-PCR for cells treated with 30 nM of NTC or pro-A1B ASO for 24 h with and without poly (I:C). (**G**,**H**) qRT-PCR for *HNRNPA1* and ISG with overexpression of myc-hnRNP A1 or myc-hnRNP A1B cDNA.

To evaluate whether the loss of hnRNP A1 is critical or whether the ratio between *HNRNPA1*-encoded isoforms is also important, we developed a splice-switching oligonucleotide (SSO) to promote exon 7B inclusion. We designed and screened 38 SSOs (data not shown) targeting intronic conserved elements previously reported as important to the regulation of exon 7B alternative splicing (CE1a, CE4, CE9 and CE6)^13,31^ and identified one suitable SSO that gave dose-dependent results. This SSO, named pro-A1B, base-pairs within the CE6 regulatory sequence, effectively disrupting RNA-RNA interactions and promoting exon 7B inclusion (Fig. 5A). Using RT-PCR, we first validated the dose- and time-dependent effect of the pro-A1B SSO compared to a non-targeting control (NTC) in A549 cells. The initial exon 7-7B-8/exon 7-8 ratio was successfully increased 10 to 16-fold (from 0.05 to 0.5–0.8) using pro-A1B SSO for 24–48 h in a dose-dependent manner (Fig. 5B,C). We then evaluated whether this was sufficient to induce changes at the protein level. While hnRNP A1B protein was indeed increased, hnRNP A1 protein levels were similar among all conditions (Fig. 5D). Interestingly, raising hnRNP A1B expression via treatment with 30nM of pro-A1B SSO for 48 h did not impact the expression of the selected innate immune genes (*RSAD2*, *IFIT1*, *IRF7*, *OAS1*, and *OAS2)*, neither in basal conditions nor following stimulation with the viral dsRNA mimic polyinosinic:polycytidylic acid [poly (I:C)] (Fig. 5E,F). Finally, we tested the effect of overexpression of Myc-tagged-hnRNP A1 or hnRNP A1B. In these conditions, the levels of the selected genes were relatively unchanged (Fig. 5G,H). Taken together, these results suggest that the loss of hnRNP A1 is responsible for the de-repression of a subset of the innate immune genes.

### *HnRNP* A1 cytoplasmic translocation and colocalization to RLBs following poly (I:C) challenge

**Fig. 6:**
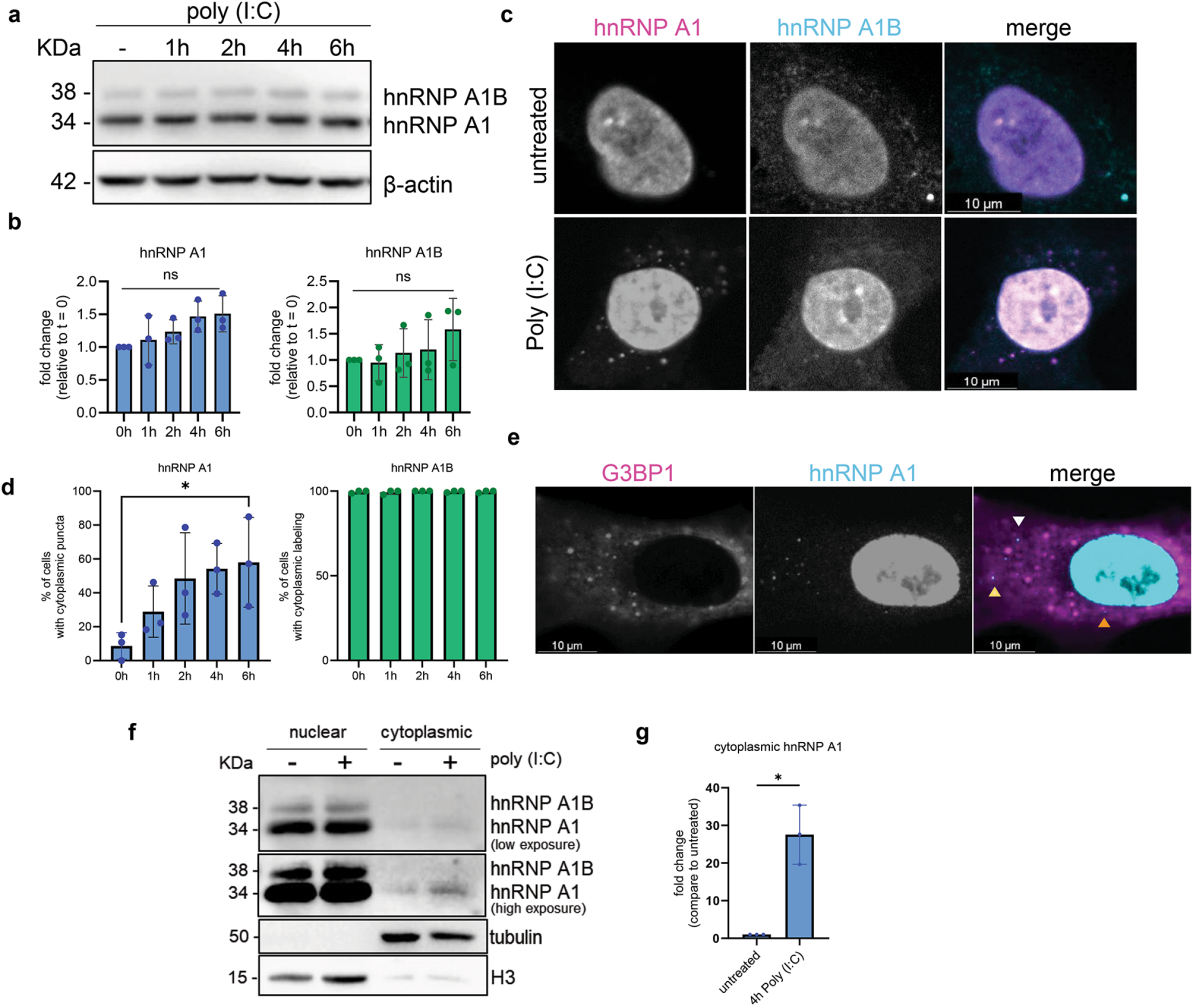
HnRNP A1 translocates to the cytoplasm and is recruited to RLBs during poly (I:C) challenge. (**A**) Western blot for hnRNP A1, hnRNP A1B and β-actin and (**B**) quantification. (**C**) Immunofluorescence for hnRNP A1 and hnRNP A1B and (**D**) quantification of the number of cells with cytoplasmic puncta. (**B**,**D**) (**E**) Immunofluorescence for G3BP1 and hnRNP A1 after 6 h of poly (I:C) treatment (yellow arrowhead: colocalized with G3BP1, orange arrowhead: in close proximity, white arrowhead: does not colocalize). (**F**) Western blot of the nuclear/cytoplasmic fractionation for hnRNP A1/A1B, tubulin. (**G**) Quantification of cytoplasmic hnRNP A1. Kruskal–Wallis test was used for statistical significance.

The increased level of dsRNA sensors, as well as other players involved in the antiviral response, when hnRNP A1 is depleted led us to question whether the level and/or location of either isoform changes when cells are exposed to dsRNA. To test this, A549 cells were treated with poly (I:C) for 1, 2, 4–6 h which did not change the protein levels of either isoform (Fig. 6A,B). We also examined the cellular localization of hnRNP A1 and hnRNP A1B by immunofluorescence using isoform specific antibodies which revealed a subtle but non-significant increase in the fluorescence intensity of hnRNP A1 and hnRNP A1B labelling in individual cells (Fig. 6C and data not shown). As previously reported^9^, in unstressed/basal conditions, hnRNP A1 is primarily located in the nucleus while hnRNP A1B localizes to both the nucleus and cytoplasm (Fig. 6C,D). During treatment with poly (I:C), the number of cells displaying hnRNP A1 cytoplasmic puncta increased while hnRNP A1B expressing cells remained largely unchanged (Fig. 6C,D). Of note, it has been previously reported that A549 cells form RNase L bodies (RLBs) in response to dsRNA, a stress response that differs from canonical stress granules by the activation of RNase L occurring either before or simultaneous with the activation of PKR^32–34^. Indeed, we observed that some of the cytoplasmic hnRNP A1 puncta co-localized with G3BP1 (yellow arrowheads), which is reported to label RLBs^34^ (Fig. 6E). Co-labeling of G3BP1 and the RLB-specific marker DHX9, but not the stress granule marker TIA-1, confirms that the observed cytoplasmic puncta are RLBs (Fig. S3). Other hnRNP A1 cytoplasmic puncta were found in close proximity (orange arrowhead) of G3BP1 puncta or not (white arrowhead). HnRNP A1 is known to translocate to the cytoplasm during stress^35^. Thus, we performed a nuclear-cytoplasmic fractionation of A549 cells untreated or stressed with poly (I:C) for 4 h. Immunoblotting indicated that hnRNP A1 protein levels were higher in the cytoplasmic fraction during poly (I:C) challenge compared to untreated cells (Fig. 6F,G). Taken together, dsRNA stress triggers hnRNP A1 translocation to the cytoplasm and partial recruitment to RLBs.

### Depletion of hnRNP A1 but not hnRNP A1B enhances the interferon antiviral response via PKR.

Next, we wanted to determine if the loss of hnRNP A1/A1B has a functional impact on the antiviral response by modulating the activity of the effectors RNase L and PKR. The OAS enzymes, which our data indicate to be repressed by hnRNP A1, are intracellular sensors of dsRNA that synthetize 2’, 5’-oligoadenylates (2–5 A) when dsRNA is recognized. These 2–5 A molecules bind the RNase L (monomer) which is subsequently activated upon dimerization, resulting in the cleavage of viral and cellular RNA, including circular RNA (circRNA)^36,37^. The degradation of circRNA, as well as binding to dsRNA, is required for the activation of PKR by autophosphorylation in early cellular innate immune response^37^. Using siRNA, we depleted either both isoforms (siA1/A1B) or uniquely hnRNP A1B (siA1B#1) from A549 cells (Fig. S4A) and then challenged with poly (I:C) for 1–4 h. Activation of the catalytic activity of RNase L was assessed by looking at the RNA integrity number (RIN). Before treatment, RIN was > 9.4 in all three conditions, confirming that elevated ISG do not trigger activation of the pathway without stimuli. As expected, the RIN decreased upon dsRNA-mediated activation of the pathway in a time-dependant matter (Fig. S4B). A closer look at the fold change of the RIN, showed a small but significant decrease after 1 h of stress only in siA1/A1B cells, but not siA1B cells, possibly suggesting that RNase L activation is accelerated when hnRNP A1 protein levels are decreased.

**Fig. 7:**
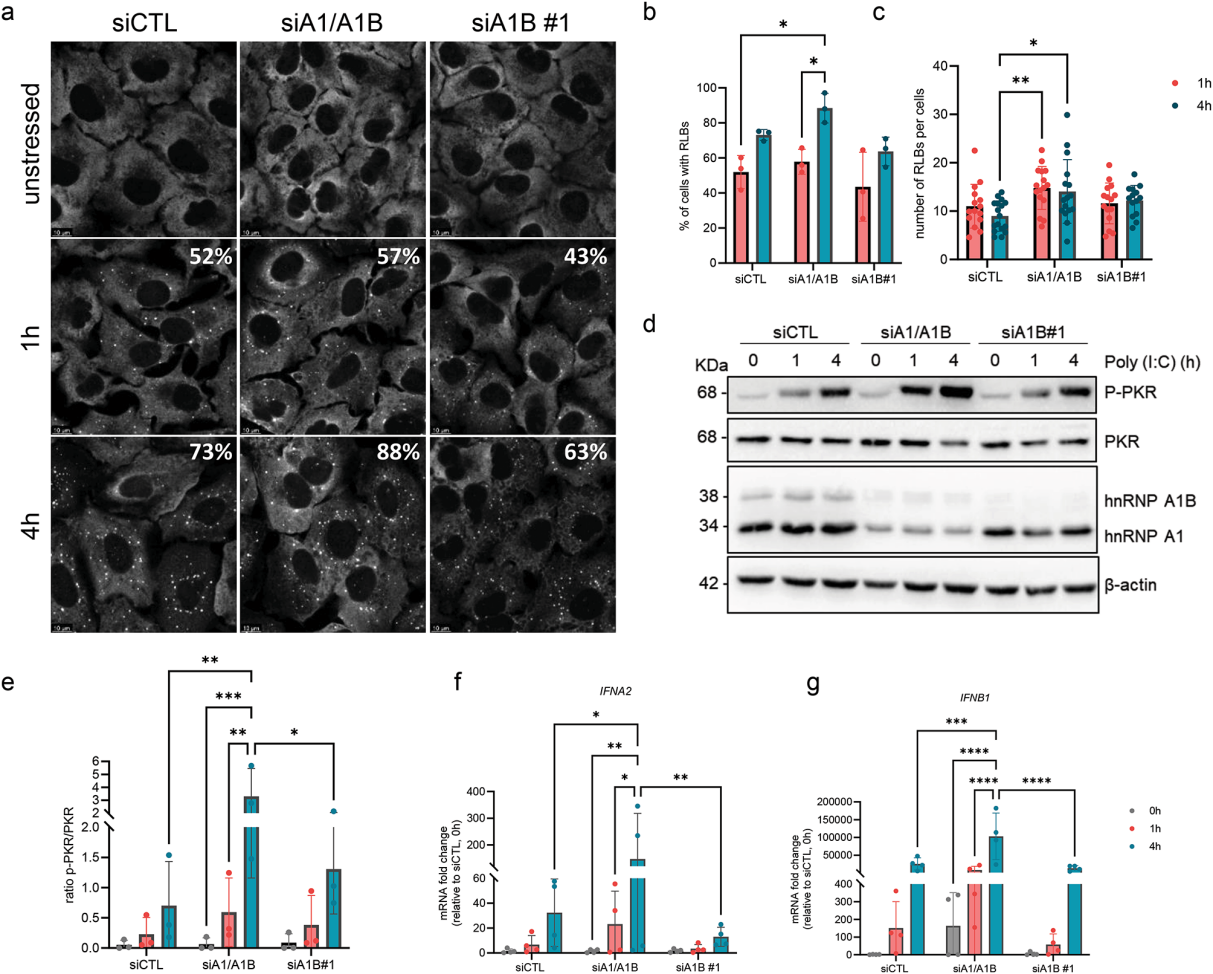
Depletion of hnRNP A1 increase PKR phosphorylation and interferon level during dsRNA stress. (**A**) Immunofluorescence for G3BP1 in siCTL, siA1/A1B and siA1B#1 cells in unstressed, 1 and 4 h of poly(I:C) conditions. Quantification of the (**B**) percentage of cells with RLBs and (**C**) the number of RLBs by cell. (**D**) Western blot for hnRNP A1/A1B, p-PKR, PKR and β-actin. (**E**) Ratio of p-PKR/PKR was quantified. qRT-PCR for (**F**) *IFNA2* and (**G**) *IFNB1*. Two-way ANOVA was used for statistical significance.

Quantification of RLB formation, using G3BP1 as a marker, also showed differences consistent with the idea that the activation of this pathway is accelerated (Fig. 7A). Indeed, the percentage of cells with RLBs was significantly increased between 1 and 4 hours only in siA1/A1B treated cells (57% at 1 h, 88% at 4 h), but not in siCTL (52% at 1 h, 73% at 4 h) and in siA1B#1 (43% at 1 h, 63% at 4 h) (Fig. 7A,B). The number of RLBs per cell was also higher in siA1/A1B versus siCTL after 4 hours of poly (I:C) (Fig. 7C) and the mean size of individual RLBs was increased at 4 hours in siA1/A1B compared to siA1B only (Fig. S4C). Taken together, these data suggest that PKR activation is increased when hnRNP A1 is depleted. Indeed, immunoblotting indicate that while PKR phosphorylation is induced by poly (I:C) treatment in siCTL cells as expected, it is increased 4.7-times more in siA1/A1B cells, while the activation in siA1B cells is comparable to siCTL cells (Fig. 7D,E). Thus, PKR is more activated when hnRNP A1 protein levels are decreased. Consistent with reports that RLB formation amplifies interferon type I signaling^38^ and that we also have an elevated level of IRF7, a critical transcription factor for the induction of IFN-α/β^39^, we observed robust increases in *IFNA2* (146-fold) and *IFNB1* (1.9 × 10^4^-fold) after 4 hours of poly (I:C) stress in hnRNPA1/A1B depleted conditions, compared to untreated siCTL cells (Fig. 7F,G). We attribute this accelerated and exaggerated response to hnRNP A1 depletion since siA1B cells displayed *IFNA2* and *IFNB1* induction that was comparable to siCTL cells, both in terms of kinetics and levels. In summary, our data indicate that hnRNP A1 represses signaling relevant to the dsRNA-mediated interferon response.

## Discussion

HnRNP A1 is an RNA binding protein which has dynamic spatiotemporal regulation and is reported to be a key regulator of multiple cellular functions including transcriptional regulation, splicing, telomere maintenance, mRNA transport, protein translation and miRNA biogenesis^3^. Most studies on *HNRNPA1* have focused on the shorter isoform hnRNP A1, with relatively little attention paid to the longer isoform hnRNP A1B or toward understanding the functional differences between isoforms. To assess the cellular impact of each *HNRNPA1-*encoded isoform without confounding effects of the other isoform, we employed a cell line in which the endogenous *HNRNPA1* gene has been inactivated (parental CB3), as well as two stable lines in which the expression of hnRNP A1 and hnRNP A1B were independently reconstituted to similar levels. Evaluation of the consequences of the sole expression of one isoform over the other on total gene expression was assessed by bulk RNA-sequencing, revealing both common and distinct effects on the transcriptome, compared to the parental line. It has been previously reported that even though CB3 cells have been reconstituted to express hnRNP A1 isoforms, other hnRNPs may have compensated for it in various cellular functions^40^. For this reason, we also performed a direct comparison of the CB3 A1 and CB3 A1B transcriptomes to gain a better perspective of which transcripts may be regulated by each isoform. Surprisingly, our strongest functional enrichment was related to innate immunity including dsRNA detection, virus infection, and interferon response. A closer inspection indicated that most of the genes in this cluster were downregulated by hnRNP A1 expression compared to hnRNP A1B, leading us to hypothesize that hnRNP A1 acts as a transcriptional repressor of ISG genes. We acknowledge that we did not directly investigate whether the regulation of differentially expressed genes by hnRNP A1 and/or hnRNP A1B occurs through direct binding to the RNA. While understanding the mechanism by which hnRNP A1/A1B regulates their different targets is important to better understand their function(s), the current publicly available CLIP-seq datasets for hnRNP A1 either use the monoclonal antibody hnRNP A1 (4B10), which primarily detects the hnRNP A1 isoform, or relies on inducible tagged-hnRNP A1 expression, and thus neither effectively include hnRNP A1B. Consequently, no CLIP-seq data specific to hnRNP A1B is currently available, making it challenging to perform a meaningful Gene Set Enrichment Analysis that correlates direct RNA binding with our RNA-seq results. As mentioned previously, hnRNP A1 has multiple functions in RNA metabolism including splicing, transcription and stability. Splicing analyses (data not shown) did not indicate robust changes in alternative splicing of the genes that were differentially expressed in the RNA-seq datasets presented here, confirming that the changes in gene expression are likely not due to abnormal splicing. Furthermore, given that hnRNP A1 is primarily nuclear, while canonical RNA degradation occurs mainly in the cytoplasm, it is unlikely that hnRNP A1 directly promotes mRNA degradation of selected ISGs in basal conditions. Further experiments will be needed to dissect the underlying mechanism(s) by which hnRNP A1 regulates the expression of these ISGs.

**Fig. 8:**
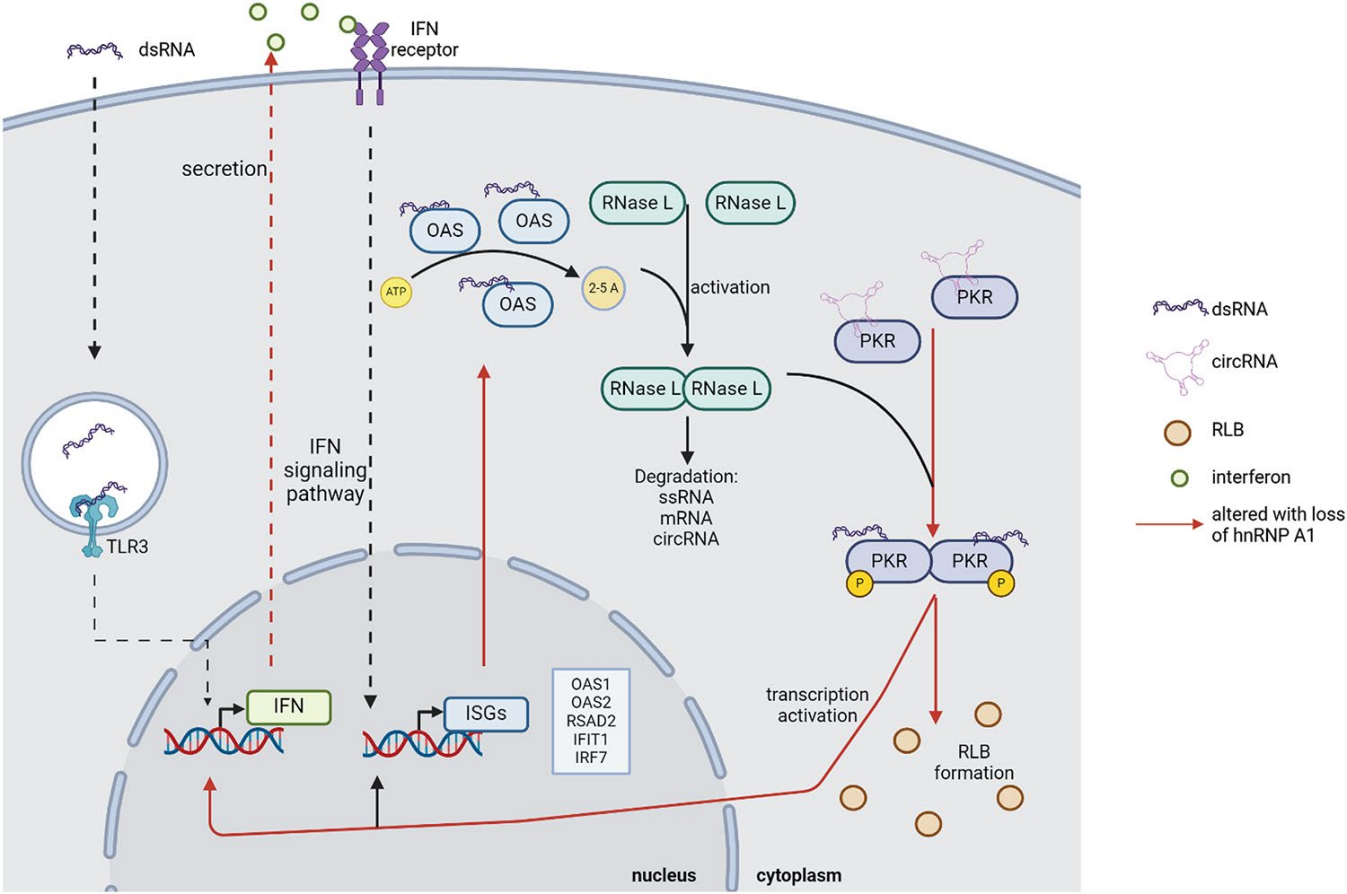
The Interferon-Induced OAS-RNase L/PKR Pathway and the effect of hnRNP A1 depletion. DsRNA are recognized by TLR3 and activate signaling pathway to induce type I interferon. Secretion of IFN activates the IFN receptor and subsequent signaling pathway, ultimately leading to transcription of ISGs, including the dsRNA sensors OASs. Upon binding to dsRNA, OASs will synthesize 2–5As, second messenger needed for the dimerization and activation of RNase L. RNase L will degrade RNA, including circRNA, which maintains PKR in an inactivated state. At the same time, PKR will also bind dsRNA and will be activated following an autophosphorylation event. This facilitates the formation of RLBs and results in the activation of transcription of IFNs and ISGs. Arrows in red represent the various steps modulated by hnRNP A1 depletion. Created in BioRender. Vande Velde, C. (2025) https://BioRender.com/c96y999.

Since this omics study was performed on mouse erythroleukemia cells, we opted to use a human cell line (A549) to explore the broader relevance of the hit genes. Indeed, depletion of both isoforms or of only hnRNP A1B in these cells confirmed that hnRNP A1 represses the expression of selected ISGs in a human context, except for *OAS*3 (Fig. 8). Using poly (I:C), we effectively mimicked viral infection and/or elevated intrinsic dsRNA. For the duration of the experiment, we did not observe robust changes in protein level, however a portion of hnRNP A1 translocated to the cytoplasm and co-localized with G3BP1-positive RNase L puncta during the challenge. Since expression of OAS family members were elevated in our transcriptomics data, we explored the consequences of the loss of hnRNP A1 on the effectors of the dsRNA-mediated interferon antiviral response, namely RNase L and PKR. Since transcripts encoding OAS family members were detected at higher levels following hnRNP A1 depletion, we expected strong activation of RNase L^41^. However, in this cell system, we only observed a modest decrease in RIN, indicative of RNase L function, compared to the control. This observation is consistent with a report that OAS3 has been shown to be the primary mediator of RNase L activation in A549 cells in response to poly (I:C), with OAS1 and OAS2 having a lesser effect^42^. Moreover, while all OASs synthetize 2–5 A, the second messenger that activates the cleavage activity of RNase L, they have been shown to have cell- and virus-type specific activation. Specifically, upon infection with West Nile, Sindbis, Influenza, or Vaccinia viruses, OAS3 plays the dominant role in RNase L activation, while OAS2 fulfills that role in the context of Zika virus infection^42,43^. Similarly, an example of cell type specificity is the observation that SARS-CoV-2 infection stimulates *OAS2* expression in neuronal, but not lung, organoids^44^. Dysfunction of the OAS-RNase L pathway has also been reported in many disease contexts, including cancer and neurodegenerative disease^36,42^. For this reason, despite the small change in RNA decay observed in our study, it remains possible that hnRNP A1-mediated regulation of the activators of RNase L in a different cellular context, e.g. one where OAS1/2 are the primary participants. Nevertheless, higher levels of ISGs in basal conditions was sufficient to increase PKR phosphorylation. This phosphorylation of PKR is associated with the activation of interferon regulatory factor 3 (IRF3)^45^, a critical transcription factor for type I interferons that works with IRF7. Our data show that IRF7 and the downstream expression of type I interferons (*IFNA2* and *IFNB1*) is elevated in hnRNP A1 depleted condition.

Due to the similarity of the sequence between the two isoforms of *HNRNPA1*, we were unable to uniquely target the shorter hnRNP A1 isoform using siRNA. However, changing the isoform ratio using SSO, or uniquely overexpressing hnRNP A1 or hnRNP A1B, did not change the selected ISGs in basal conditions, similar to siA1B conditions. Combined with the results obtained using the CB3 cell line, in which the endogenous *HNRNPA1* locus has been inactivated, and the two derivative cell lines where each isoform were separately reconstituted to comparable levels (CB3 A1 and CB3 A1B), we conclude that hnRNP A1 is the isoform mediating the observed effect.

DsRNA species exhibit a pathogen-associated molecular pattern that is recognized by dsRNA sensors, triggering type I interferon response. They can derive directly from the viral genome or be self-derived^46^. Many RBPs have been implicated in the control and recognition of dsRNA, including TDP-43 and hnRNP C, which can restrict the accumulation of self-derived dsRNA and modulate the interferon pathway via RIG-1^47,48^. Neurons have elevated levels of self-derived dsRNA^49^ and high levels of dsRNA have been suggested as potentially neuroprotective by preventing viral infection^49^. However, this process must be fine-tuned to ensure that protection against viruses does not trigger cell death or excessive neuroinflammation^49^. The collaboration of multiple RBPs is essential for maintaining this balance and we have identified hnRNP A1 as a new player in the regulation of the dsRNA response.

## Materials and methods

### Cell culture and transfection

Mouse erythroleukemia CB3 parental (CB3) (Gift from Dr. Benoit Chabot) and stable transfectants (CB3 A1 and CB3 A1B) cells were cultured in Cytiva HyClone™ Minimum Essential Medium (MEM) alpha modification (GE healthcare) supplemented with 10% of fetal bovine serum (FBS) and 1% of penicillin-streptomycin (P/S). Media for stable transfectants was also supplemented with geneticin (G418) to a final concentration of 400 µg/ml for selection. A549 (Gift from Dr. Nathalie Grandvaux) cells were cultured in Ham’s F-12 Nutrient Mix (Gibco) supplemented with 10% FBS and 1% P/S. A549 were transfected with ON-TARGETplus siRNA (400 pmol) (Dharmacon) using Oligofectamine™ (Invitrogen) for 72 hours. The following siRNA were used: siCTL; ON-TARGETplus Non-targeting Control siRNAs #1, siA1/A1B ; 5’-AAUGAGAGAUCCAAACACCAAUU-3’, siA1B#1 ; 5’-GCUAUGACAGCUAUAACAACGUU-3’, and siA1B#2; 5’-GCAGCUAUGACAGCUAUAACAUU-3’. Cells were transfected with *p*oly (I:C) (1 µg/ml) (Sigma) using Lipofectamine™ 2000 (Invitrogen) and incubated for 1, 2, 4–6 h at 37 °C. For cDNA transfection, pCMV-SV40, pCMV-Myc-A1 or pCMV-Myc-A1B were transfected using Lipofectamine™ LTX with PLUS reagent (Invitrogen) for 24 h. Splice-switching ASO were transfected using Lipofectamine™ RNAiMAX for 24–48 h.

### RNA-seq library preparation and sequencing

Total RNA was extracted from CB3, CB3 A1 and CB3 A1B cells (in triplicate) using TRIzol™ Reagent (Invitrogen) according to the manufacturer’s instructions. RNA extracts were purified and concentrated using the RNeasy MinElute cleanup kit (Qiagen) following the manufacturer’s instructions. Library preparation and sequencing were performed by the Genome Québec Expertise and Service Center. RNA integrity numbers (RIN), measured on a Bioanalyzer (Agilent), were equal or above 9.7 for all/most samples. For two samples, the Bioanalyzer failed to calculate a RIN number, but manual inspection of the graphs confirmed quality similar to the other samples. An rRNA-depleted stranded (HMR) library was prepared and sequenced via 100 bp paired-end reads on the Illumina NovaSeq 6000 system.

### RNA-seq data analysis

Sequence reads were aligned on the *Mus musculus* reference genome mm10 using STAR aligner. Quality control was assesses using the modular tool MultiQC (v1.7)^50^ which regroups data from Samtools (1.9), QualiMap (2.2.2c) and FastQC (0.11.8). It was noted that even though all samples were of good quality, the number of aligned reads indicated that most genes had low coverage for the sample CB3_A1B_1. For this reason, this sample was excluded from subsequent analyses. Counts matrix were obtained using featureCounts^51^. Sashimi plot were generated with Integrative Genomics Viewer (IGV)^52^. Differential expression analysis was done using the DESeq2 package (3.12 release)^26^ on R (4.3.2) using a p-value < 0.05 and a Log2FoldChange) > = 1 as a significant threshold. For data visualization, ggplot2 (3.4.4) and complexHeatmap packages were used for the generation of the volcano plots and the heat maps.

### Gene ontology analysis

The functional annotation clustering tool from the Database for Annotation, Visualization and Integrated Discovery (DAVID) (v.6.7)^27^ was used for Gene ontology analysis. The *Mus musculus* genome was used for the reference background and clusters with an enrichment score ≥ 1.3 (equivalent of minus log scale of a p-value ≤ 0.05) were kept.

### Characterization of HnRNP A1/A1B (254) antibody

Polyclonal antibodies targeting hnRNP A1 and hnRNP A1B were commercially generated by Medimabs (Montréal) and previously described^9^. We demonstrated that the antibody hnRNP A1/A1B (rb254) recognize both isoforms to similar levels by direct comparison of different cell lines lysates by Western blot (Fig. S5A). An hnRNP A1B specific antibody (rb253) has been previously characterized. We also demonstrated the specificity using affinity-purified antibody pre-absorbed with GST-tagged recombinant mouse hnRNP A1 or hnRNP A1B protein (Gift from Dr. Benoit Chabot) at 1:10 ratio in PBS-T incubated overnight at 4 °C with rotation and cleared using 4B glutathione sepharose beads (Fig. S5B).

### Splice-switching oligonucleotides

Splice-switching oligonucleotides (SSO) targeting the intronic splice site of *HNRNPA1* were designed and synthetized with a uniform 2’-O-methoxyethyl (2’MOE) modifications. Pro-A1B SSO (ISISNO 1449732) and non-targeting control (NTC) (ASO ID 439272) with the following sequence was used; pro-A1B: AGGCGGCCCCAGCTTAAA and NTC: TTAGTTTAATCACGCTCG.

### RIN, qRT-PCR and PCR

RNA was extracted using the RNeasy mini kit (Qiagen) following the manufacturer’s instructions. RIN was determined using the RNA 6000 Pico Kit on the 2100 Bioanalyzer System (Agilent). For the qRT-PCR, equal amounts of RNA were reverse transcribed with the QuantiTect Reverse Transcription kit (Qiagen). Quantitative RT-PCR was done on the QuantStudio™ 7 Real-Time PCR (Life technologies) using PrimeTime™ qPCR Probe Assays (Integrated DNA Technologies). For experiment using mouse cell lines, we used pre-designed probe assays for *ACTB (*Mm.PT.39a.22214843.g), *RPLP0* (Mm.PT.58.43894205), *IRF7* (Mm.PT.58.32394021.g), *OAS2* (Mm.PT.56a.7124473), and *OAS1a* (Mm.PT.58.30459792). We used custom probe-based assay for the detection of total *HNRNPA1* with this primer set: forward: *5*′-*AGGTTCCACAACTCTTCCATC-3*′, reverse: *5*′*-TGAGAGAT CCAAACACCAAGAG-3*′, Probe *5*′*-TGGATGCTGCCATGA-3*′. For experiment using human cell lines, we used the following pre-designed probe assays: *HNRNPA1* (Hs.PT.58.38919354), *HNRNPA1* (Hs.PT.58.458382), *OAS1* (Hs.PT.58.2338899), *OAS2* (Hs.PT.58.1915755.g), *OAS3* (Hs.PT.58.4561974), *RSAD2* (Hs.PT.58.713843), *IRF7* (Hs.PT.58.24613215.g), *IFIT1* (Hs.PT.56a.20769090.g), *IFNB1* (Hs.Pt.58.39481063.g), *IFNA2* (Hs.Pt.58.24294810.g), *18S* (Hs.PT.39a.22214856.g) and *GAPDH* (Hs.PT.39a.22214836). The relative change in expression was calculated using the 2^–ΔΔCt^ method. For PCR, we used Taq DNA Polymerase (NEB) with the following primers: HnRNPA1 exon 7 end-fwd 5′-GCTAGTGCTTCATCCAGCCA-3′ and HnRNPA1 exon 8 start-rev 5′-CCAGAGCTTCTGCCTCCAAA–3′. Original gels are presented in supplementary Fig. 6.

### Protein extraction, nuclear/cytoplasmic fractionation and Immunoblot

Protein lysates were prepared in RIPA buffer (150 mM NaCl, 50 mM Tris pH 7.4, 1% Triton™ X-100, 0.1% SDS, 1% sodium deoxycholate) with proteases and phosphatases inhibitors. Nuclear/cytoplasmic fraction were obtained using the NE-PER™ Nuclear and cytoplasmic extraction reagents (Thermo Scientific) following the manufacturer’s instructions. Protein concentration was assessed by BCA (Pierce) and equal amounts of protein were subjected to a 12.5% SDS-PAGE, transferred to nitrocellulose and blocked with 5% powdered milk in TBS-T. Membranes were incubated with the following primary antibodies: anti-hnRNPA1/A1B(rb#254) (dilution 1:1000; custom), anti-hnRNPA1B(rb#253) (dilution 1:2000; custom), anti-hnRNPA1(4B10) (dilution 1:1000; Santa Cruz, sc-32301), anti-β-actin (dilution 1:15000; MP Biomedical, 69100), anti-IRF7(H-246) (dilution 1:500; Santa Cruz, sc-9083), anti-IFIT1 (dilution 1:1000;Abcam, ab305301), anti-tubulin (dilution 1:2000; Sigma, T6199), anti-H3a (dilution 1:5000; Proteintech, 17168-1-AP), anti-PKR(YE350) (dilution 1:2000; Abcam, ab184257), and anti-Phospho-PKR(Thr451) (dilution 1:1000; Abcam, ab32036). Membranes were subsequently incubated with the appropriate HRP-conjugated secondary antibody (dilution 1:5000; Jackson ImmunoResearch). The signal was revealed with ECL (ThermoFisher Scientific). The acquisition and quantification was done on the BIO-RAD ChemiDOC MP imaging system using the Image Lab software. Original blots are presented in supplementary Fig. 6.

### Immunofluorescence

Coverslips were fixed in 4% formaldehyde/PBS, permeabilized in PBS/0.1% Triton X-100 and blocked in PBS/0.1% bovine serum albumin (BSA). They were subsequently incubated with the following antibodies diluted in PBS/0.1%BSA: anti-G3BP1 (dilution 1:200; Proteintech, 13057-2-AP), anti-G3BP1 (dilution 1:300, BD Biosciences, 611126), anti-hnRNPA1B(rb#253) (dilution 1:100; custom), anti-hnRNPA1(4B10) (dilution 1:50; Santa Cruz, sc-32301), anti-DHX9 (dilution 1:100; Proteintech, 67153-1-IG) and anti-TIA1 (dilution 1:200; Abcam, ab40693 ). The coverslips were washed once with PBS/0.1% Triton X-100 and then twice more with PBS/0.1% BSA before the incubation with the secondary antibodies; donkey anti-rabbit AlexaFluor-488 (dilution 1:500; Life Technologies) and donkey anti-mouse AlexaFluor-594 (dilution 1:500; Life Technologies). Nuclei were labelled with Hoechst 33,342 (dilution 1:10000; Thermo Scientific) and mounted with ProLong Gold antifade reagent (Invitrogen). Image acquisition was performed on a Leica TCS SP5 confocal microscope equipped with 63 × (1.25 N.A.) oil objective and the Leica Application Suite imaging software. Intensity profile and RLBs quantification was done with ImageJ software.

### Statistical analysis, graphics and schematic

Statistical significance of experiments was determined using one-way ANOVA, two-way ANOVA or Kruskal-Wallis test with GraphPad Prism software. Error bars represent the standard error of the mean (SEM). NS *P* > 0.05, * *P* ≤ 0.05, ** *P* ≤ 0.01, *** *P* ≤ 0.001 and **** *P* ≤ 0.0001. Graphics were made with GraphPad Prism software. Schematics were created in https://BioRender.com.

## Electronic supplementary material

Below is the link to the electronic supplementary material.

Supplementary Material 1

Supplementary Material 2

Supplementary Material 3

## Data Availability

RNA sequencing data generated during the current study are available in the NCBI’s Gene Expression Omnibus database repository under the series accession number GSE286814.
