# Supplementary material for: The differential impact of HNRNPA1 isoforms on gene expression and their relevance to dsRNA-mediated innate immune response
Source: Sci Rep. 2025 May 1;15:15306. doi: 10.1038/s41598-025-99031-7 (PMC12046027; doi:10.1038/s41598-025-99031-7)

Sample name	# reads	# bases	Avg. Qual.
CB3_1	51,557,588	10,414,632,776	36
CB3_2	47,629,205	9,621,099,410	36
CB3_3	69,711,649	14,081,753,098	36
CB3_A1_1	52,662,054	10,637,734,908	36
CB3_A1_2	53,373,127	10,781,371,654	36
CB3_A1_3	150,866,743	30,475,082,086	36
CB3_A1B_2	61,029,899	12,328,039,598	36
CB3_A1B_3	60,957,836	12,313,482,872	36

Supplementary Table 1: Sample details of the RNA sequencing with Illumina NovaSeq 6000

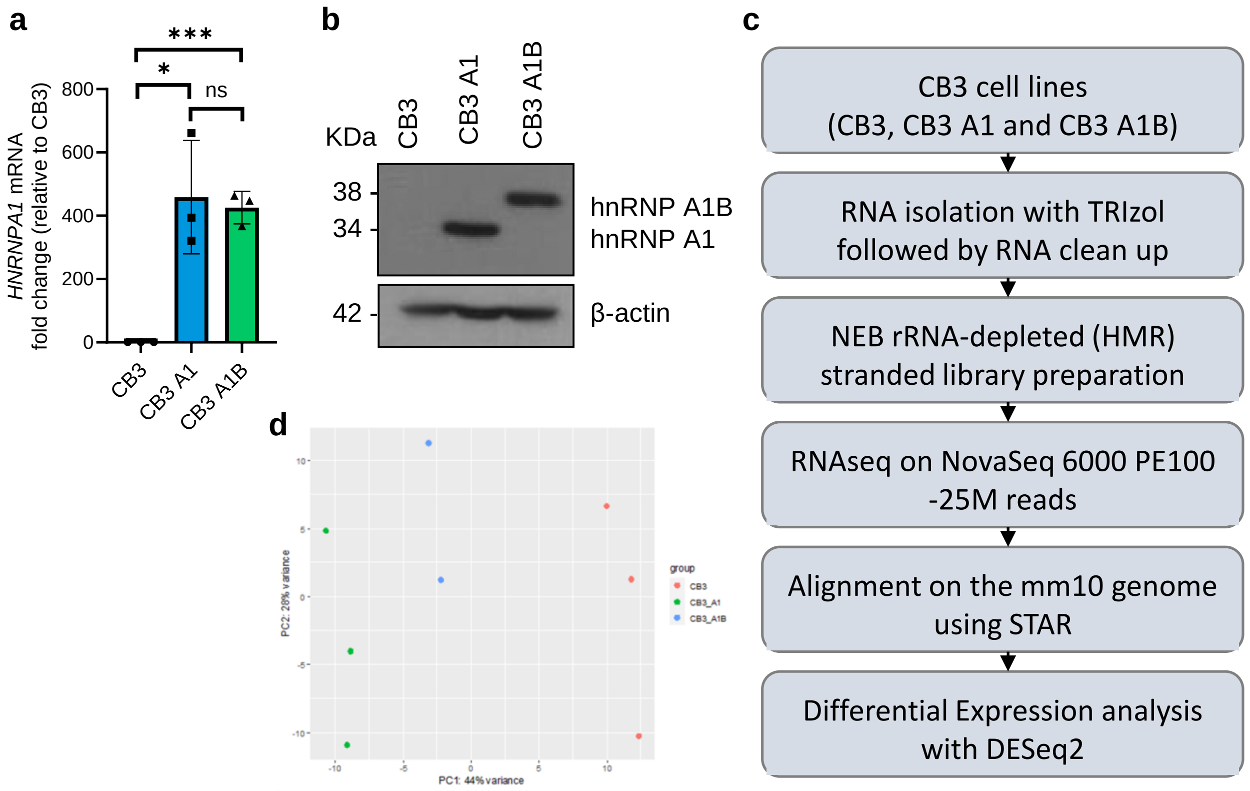

Supplementary figure 1. Validation of cell model and bioinformatics pipeline. (A) qRT-PCR for *HNRNPA1* level and (B) Western blot for hnRNP A1/A1B (custom 254 antibody) and β-actin in CB3, CB3 A1 and CB3 A1B cell lines. (C) RNA-seq pipeline. (D) Principal component analysis (PCA) plot.

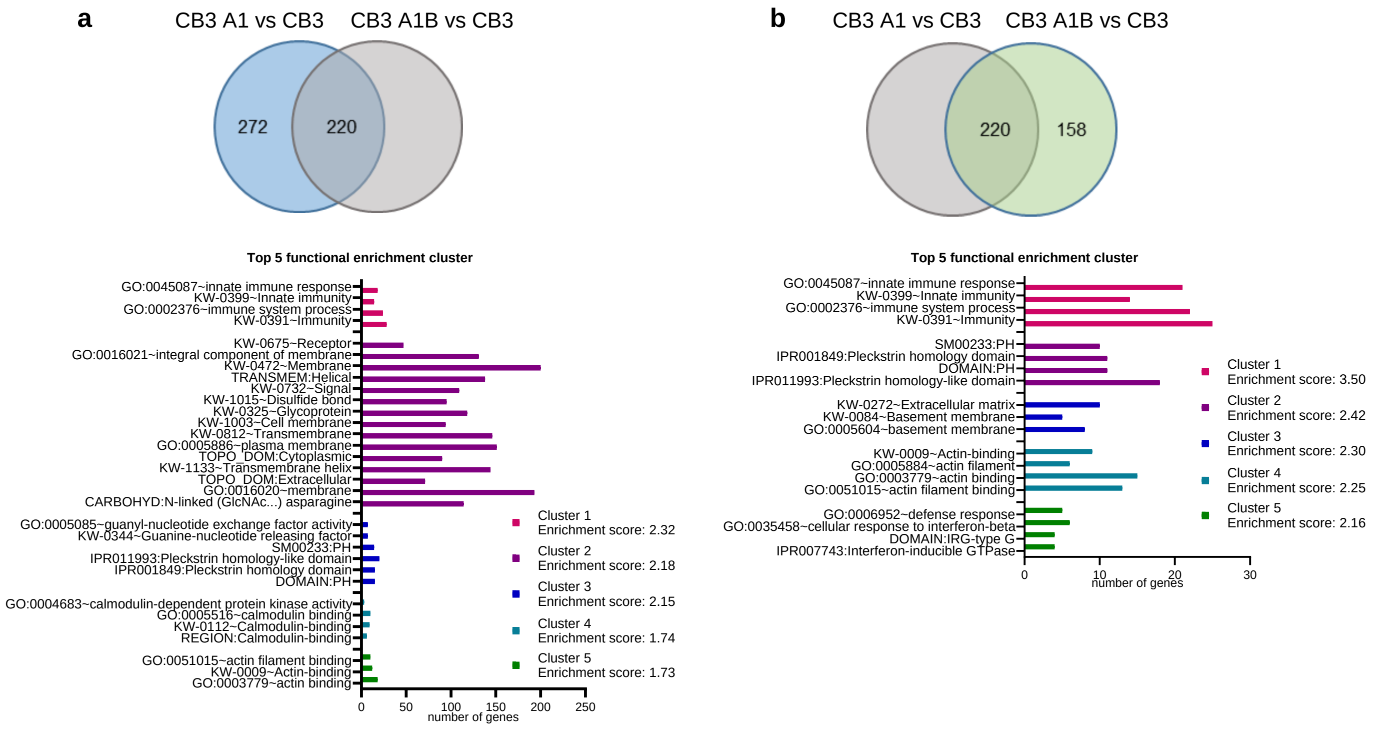

Supplementary figure 2. DAVID functional annotation clustering analysis. (A) For all genes differentially regulated in CB3 A1 vs CB3. (B) For all genes differentially regulated in CB3 A1B vs CB3.

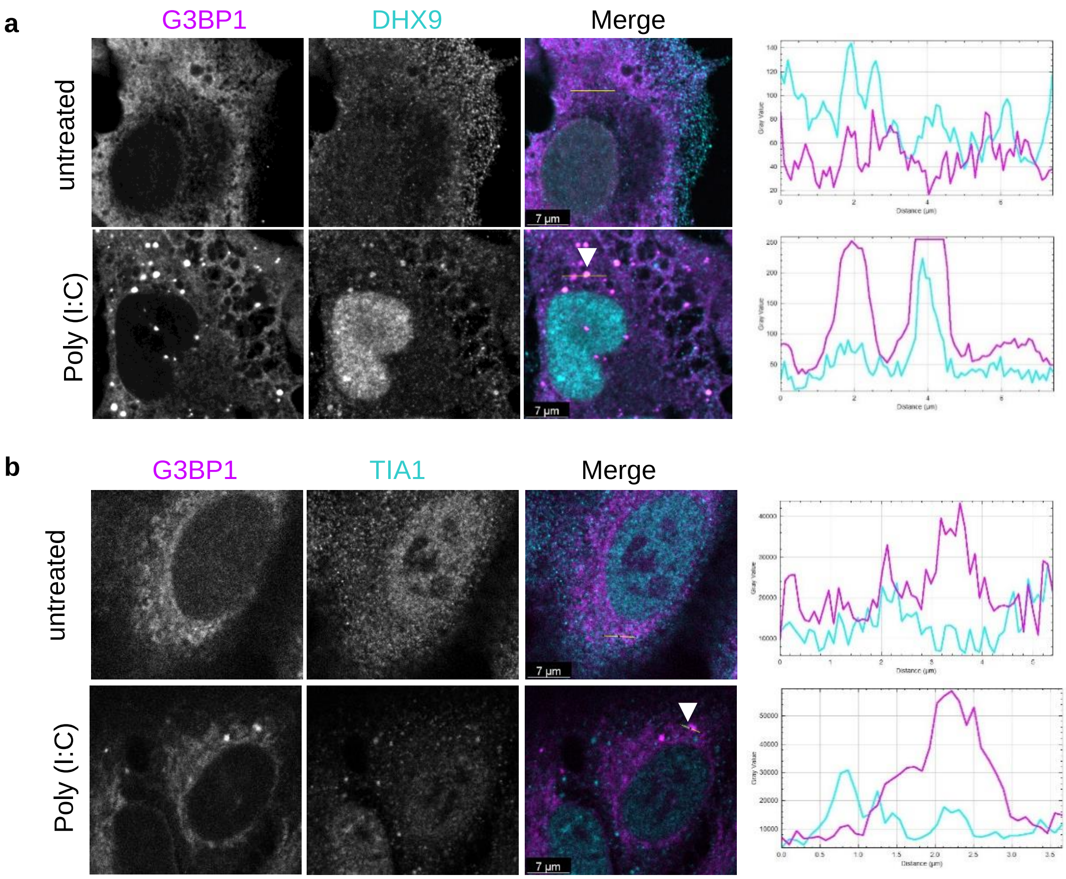

Supplementary figure 3. Validation of the formation of RLBs. (A-B) Immunofluorescence for RLBs (A) G3BP1 and DHX9; and stress granules (B) G3BP1 and TIA1.

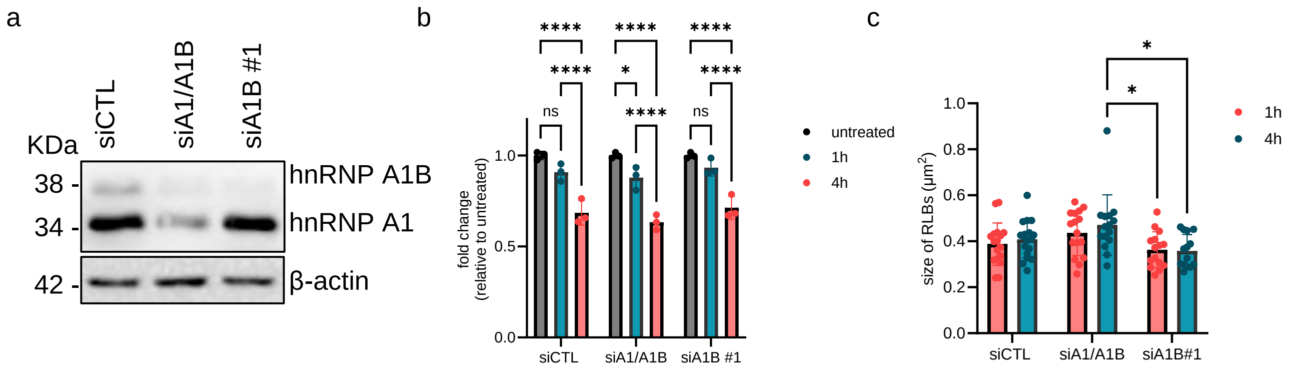

Supplementary figure 4. Effect of hnRNP A1 and/or hnRNP A1B on RIN and RLBs (A) Western blot confirming siRNA efficiency. (B) Fold change relative to siCTL t=0 for RNA integrity number (RIN). (C) Average size of RLBs.

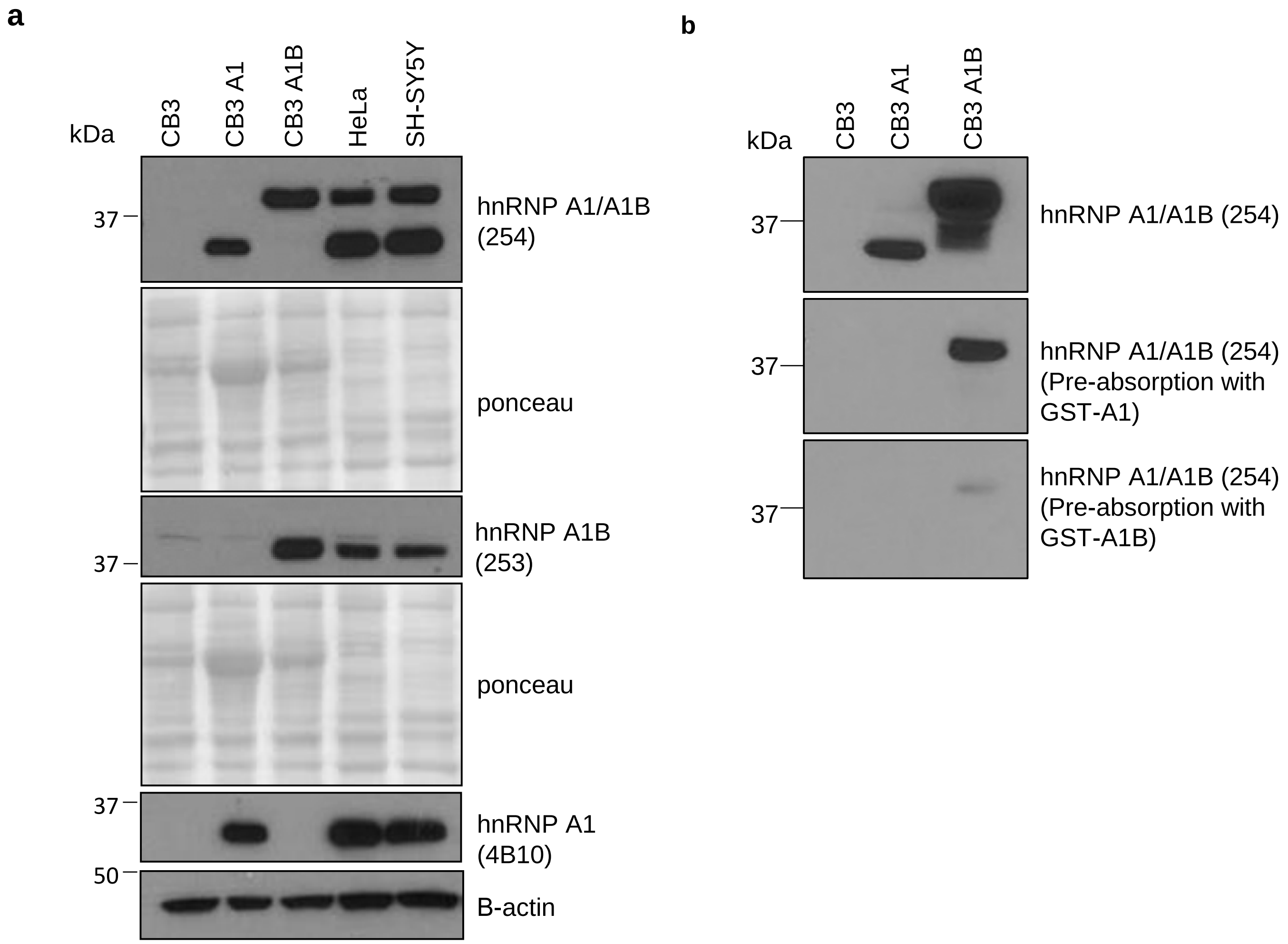

Supplementary figure 5. Validation of hnRNP A1/A1B (254) antibody. (A) Western blot using different cell lines (CB3, CB3 A1, CB3 A1B, HeLa and SH-SY5Y) and commercial (4B10) and custom (253,254) hnRNP A1/A1B antibodies. (B) Western blot without (top panel) or with pre-clearing with mouse recombinant protein GST-A1 or GST-A1B.

Supplementary figure 6. All original western blot and agarose gels. Red boxes indicate the cropped area that were included in the formatted figure.

Relevant to supplementary figure 1 b

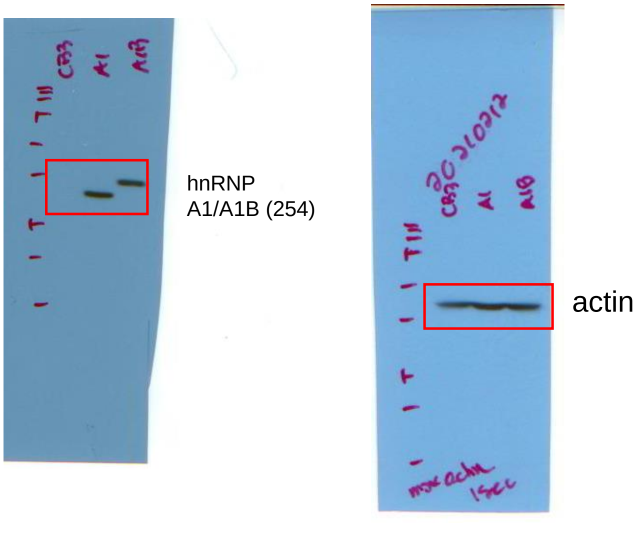

Relevant to Figure 4 a

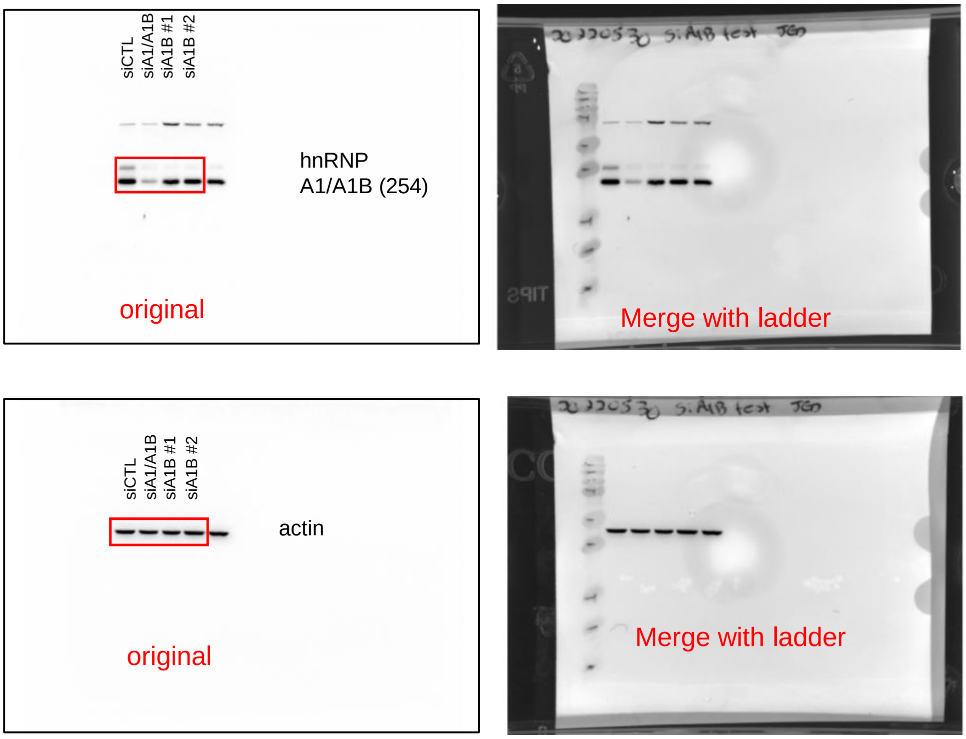

Relevant to Figure 4 j

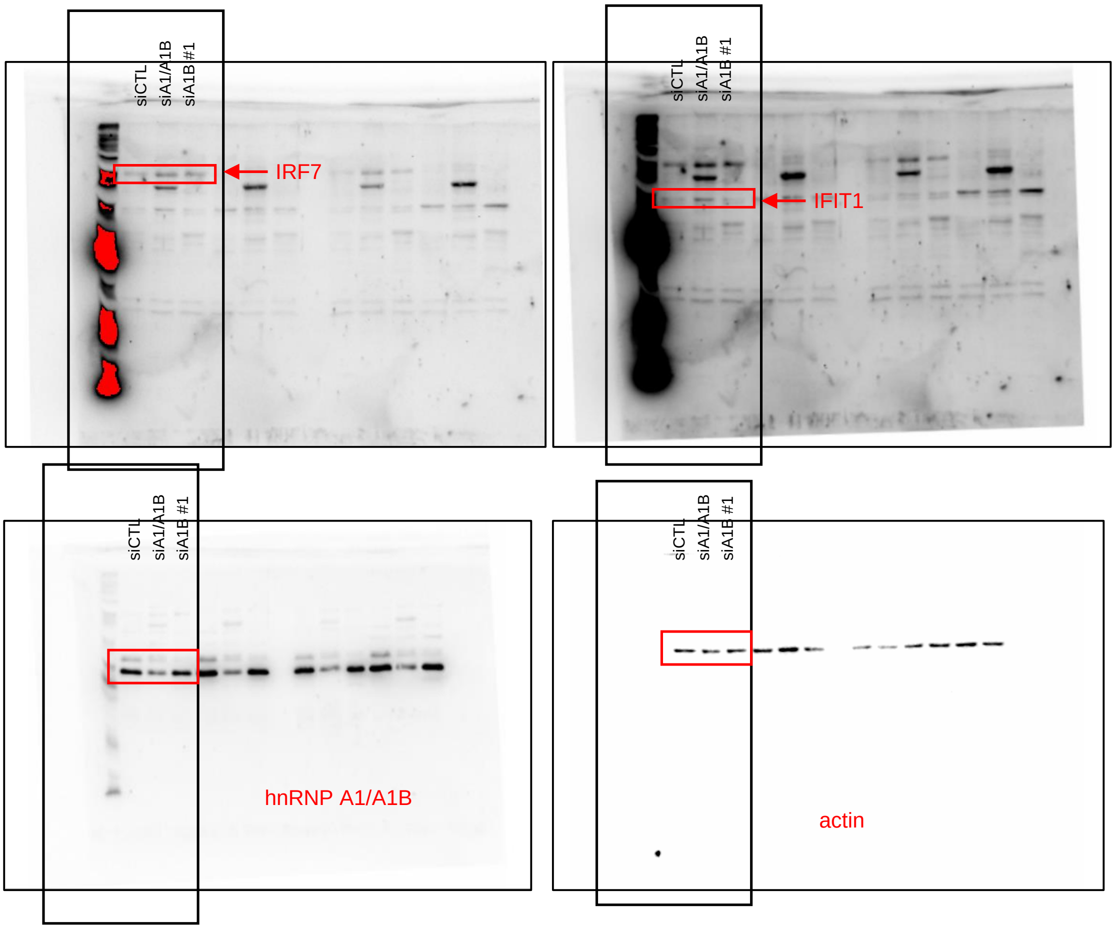

Relevant to Figure 5 b

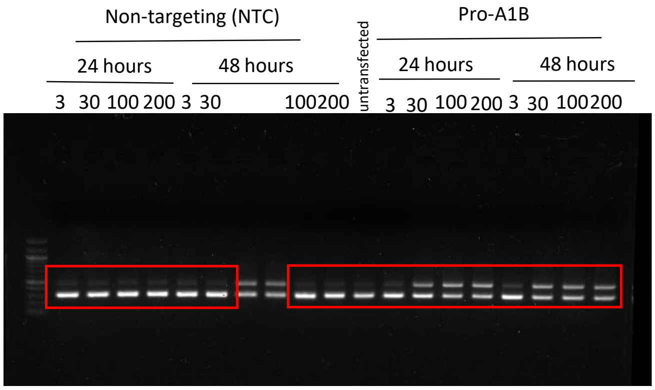

Relevant to Figure 5 d

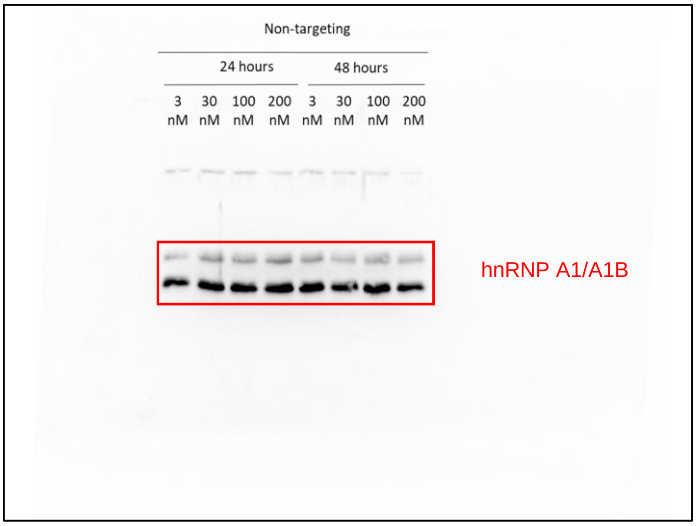

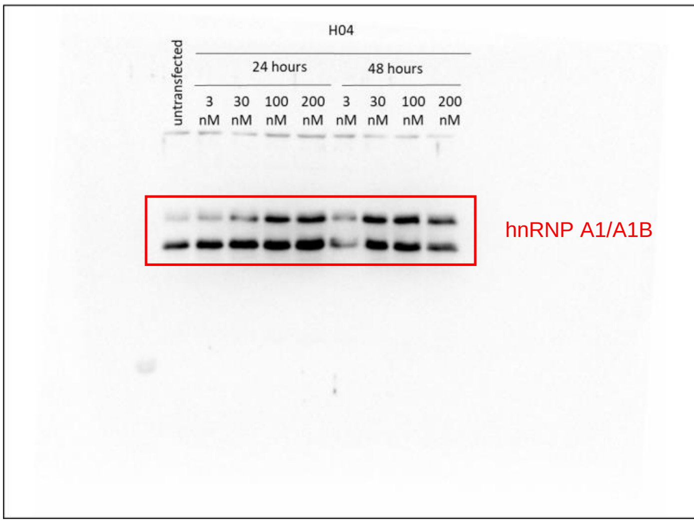

Relevant to Figure 5 e

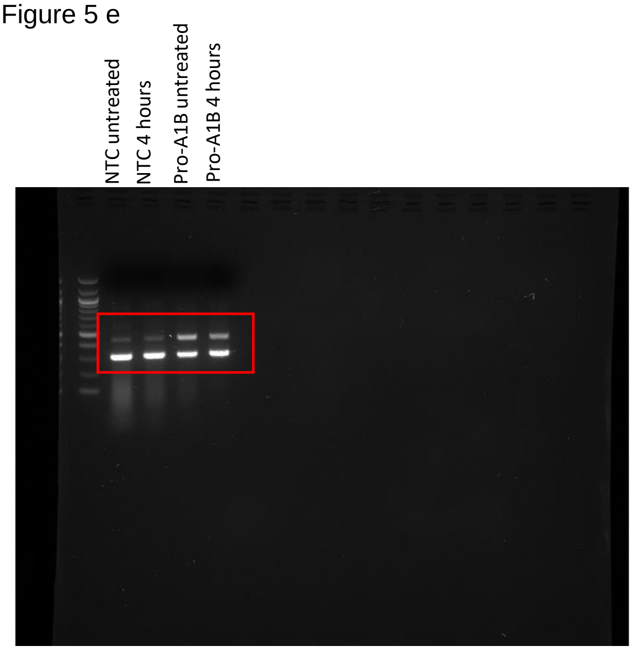

Relevant to Figure 6 a

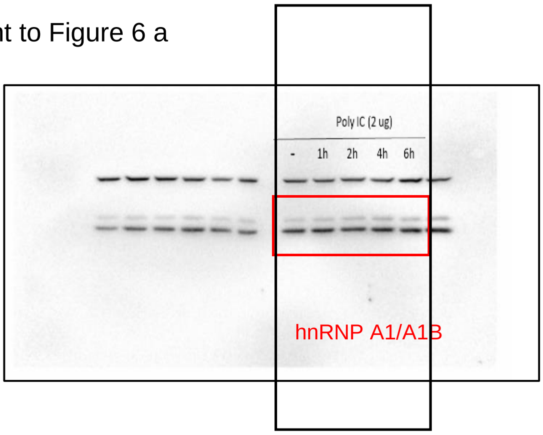

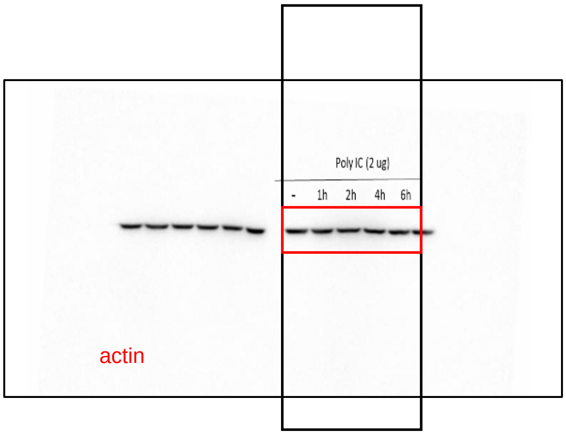

Relevant to Figure 6 f

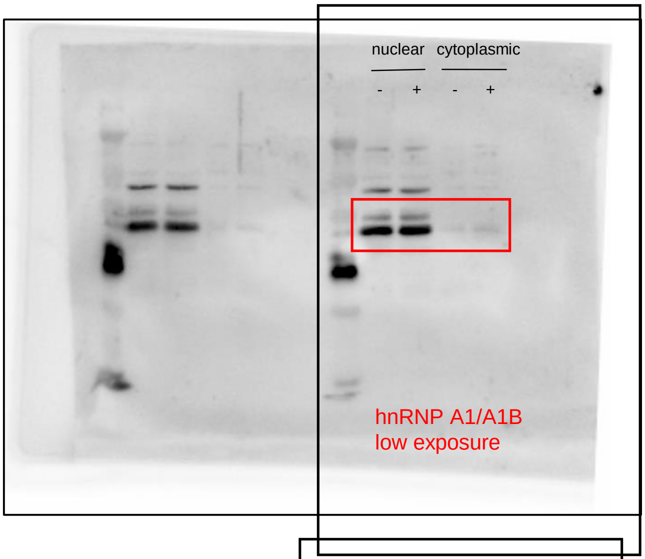

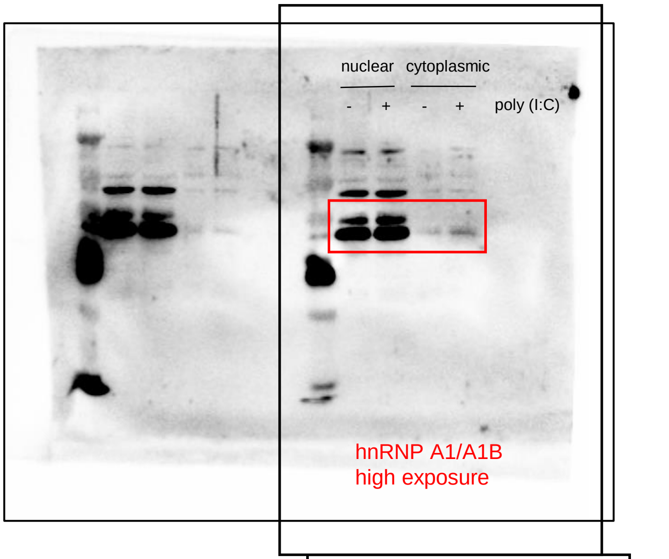

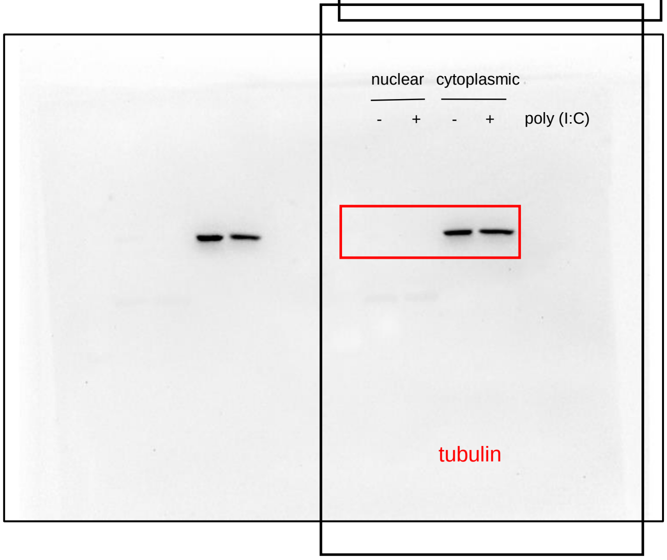

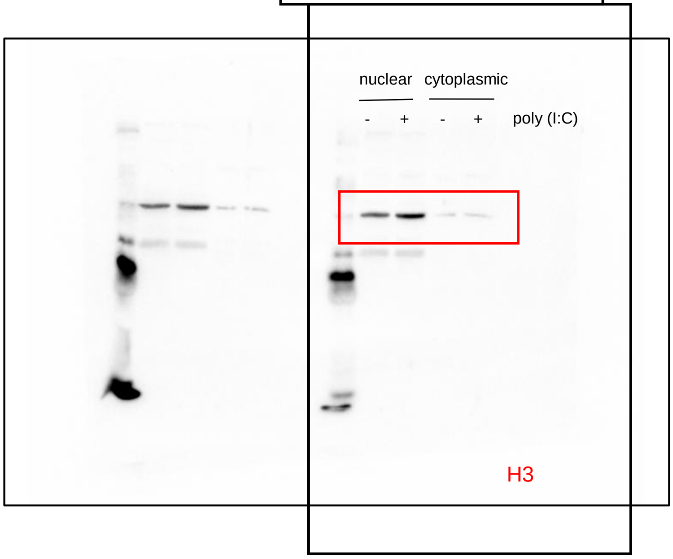

Relevant to supplemental figure 4

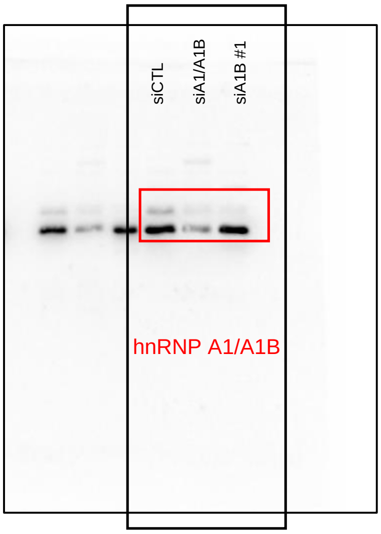

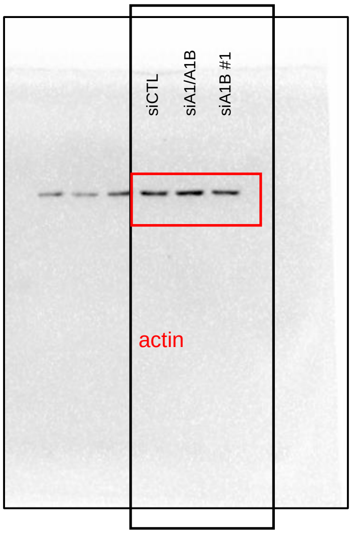

Relevant to Figure 7 D

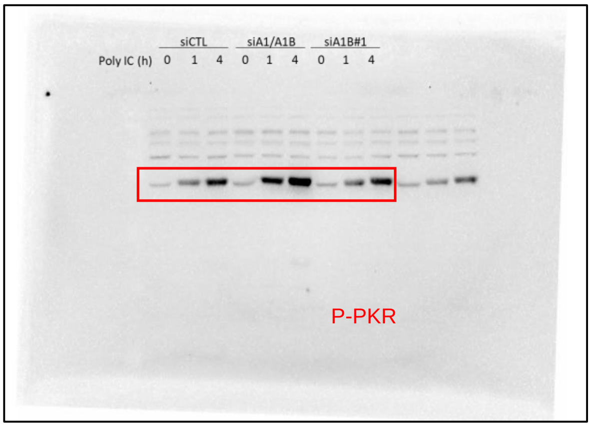

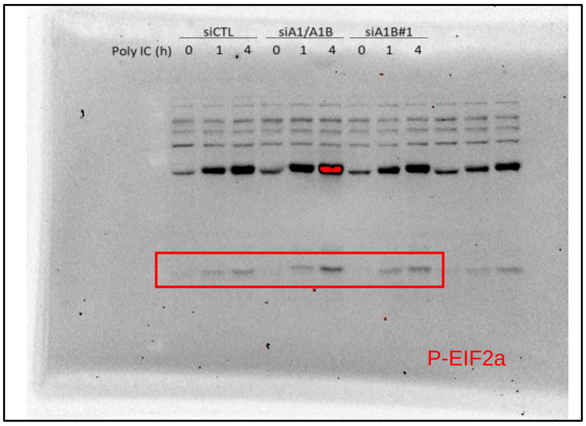

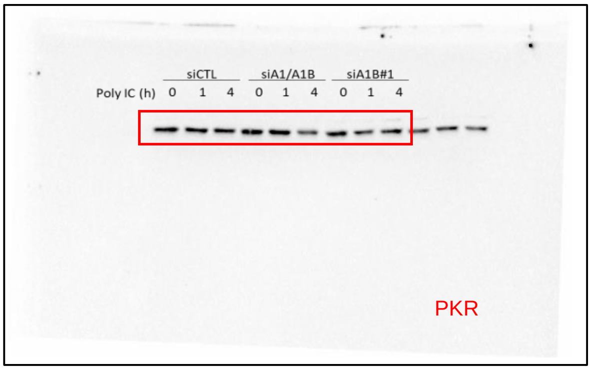

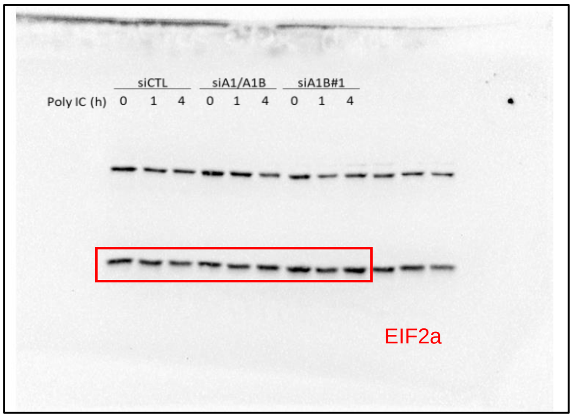

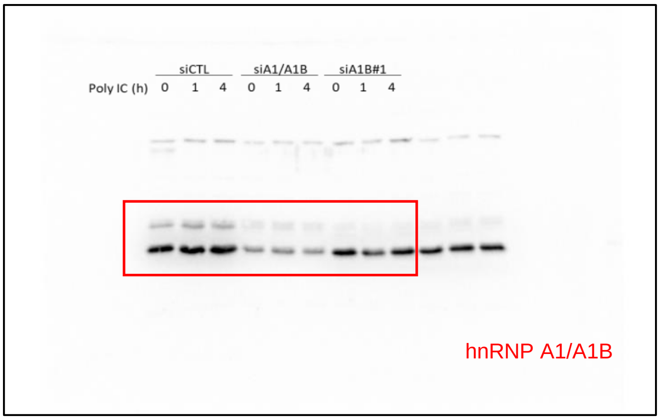

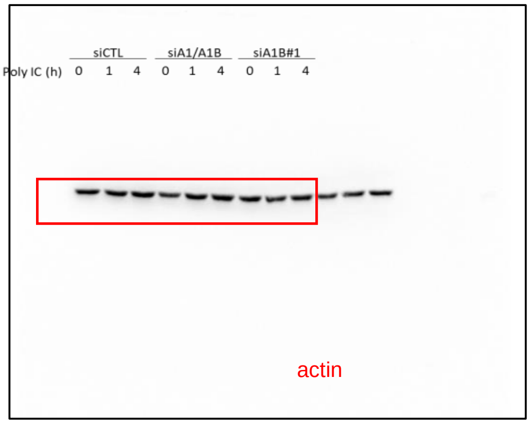

Supplementary figure 5 A

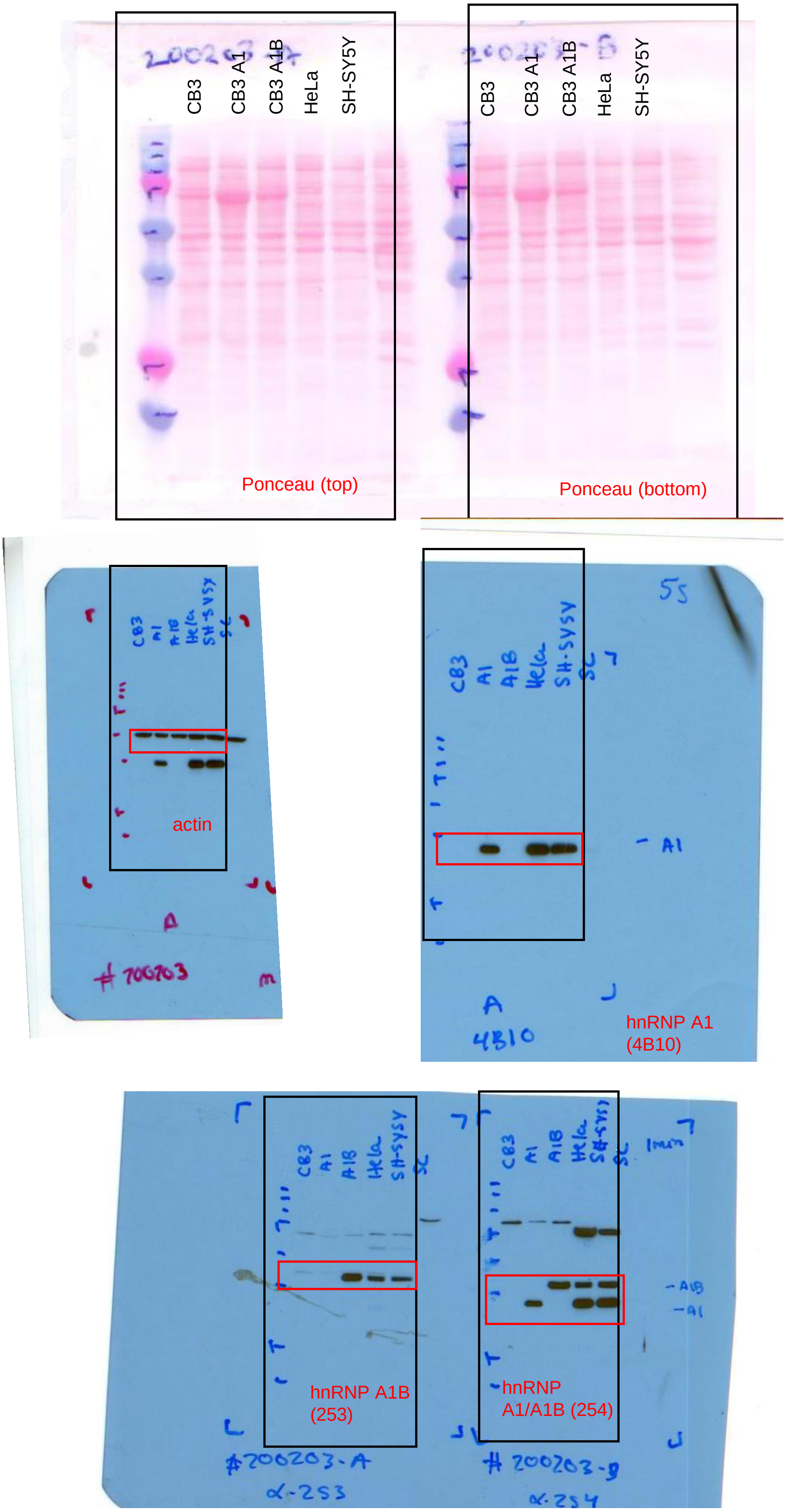

Supplementary figure 5 B

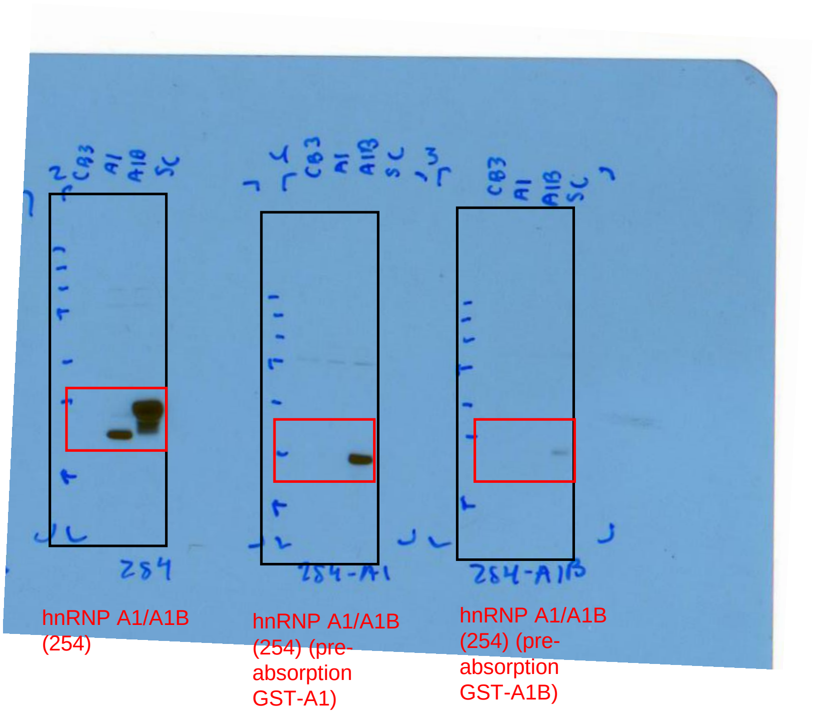

Supplement: Supplementary file 3 — Supplementary Material 3 [file 41598_2025_99031_MOESM3_ESM.pdf]
